# Aqueous extract of *Acer truncatum* leaves retards *Drosophila melanogaster* senescence by regulating amino acid metabolism and gut microbiota

**DOI:** 10.1038/s41598-025-17390-7

**Published:** 2025-10-02

**Authors:** Feng Liu, Yuchan Zhang, Lulu Zhang, Wenyu Feng, Yongkang Zhao, Jingjing Wei, Xiaoyan Zhu, Shanting Zhao

**Affiliations:** 1 College of Veterinary Medicine, Northwest A&F University, Yangling, 712100 China; 2 Chongqing YaoPharma Co., Ltd, Chongqing, 401121 China

**Keywords:** Aqueous extract, Acer truncatum leaves, Anti-aging, Microbiomes, Metabolomics, Computational biology and bioinformatics, Microbiology, Plant sciences

## Abstract

*Acer truncatum* is a unique tree species indigenous to northern China. The Chinese government approved the utilization of *Acer truncatum* leaves as a raw material for food. These leaves have been traditionally used in Inner Mongolia as a form of anti-aging medicine. However, the specific mechanism responsible for the anti-aging properties of *Acer truncatum* leaves remains unidentified. In this study, an aqueous extract of *Acer truncatum* leaves (AAL) was prepared and analyzed using UPLC-QTOF-MS/MS. the UPLC-MS/MS profile detected a total of 989 compounds in AAL, with 5 compounds of high concentration selected for quantitative analysis via UPLC-QTOF-MS/MS employing the internal standard method. Subsequently, *Drosophila melanogaster* served as a model organism to assess the impact of AAL on the lifespan and locomotor abilities. The results demonstrated a significant extension of the lifespan of *D. melanogaster* in response to AAL supplementation. Moreover, the addition of AAL to the medium enhanced the physical and anti-stress abilities of *D. melanogaster*, while preserving the integrity of their intestinal barrier. Gut microbiome analysis revealed that AAL administration positively influenced the structure and composition of gut microbes in aged *D. melanogaster*, notably reducing the prevalence of detrimental bacteria like *Enterococcus* and increasing beneficial bacteria such as *Lactococcus*. Metabolomic analysis annotated 30 potentially significant metabolites in AAL that contribute to delaying aging, predominantly associated with Phenylalanine metabolic pathways. Through a comprehensive multi-omics correlation analysis, a strong link was established between gut microbiota and metabolites following AAL treatment, highlighting how AAL prolongs the lifespan of *D. melanogaster* by modulating metabolic pathways via the gut microbiota. This study offers valuable insights into the anti-aging properties of AAL, emphasizing its ability to delay aging primarily through the regulation of metabolic pathways mediated by the gut microbiota and sets a foundation for the potential future application of AAL as a functional food.

**Supplementary Information:**

The online version contains supplementary material available at 10.1038/s41598-025-17390-7.

## Introduction

The aging process is a natural occurrence that manifests with an advancing age. It is characterized by the decline in the function of various organs and tissues of body, leading to the decrease in mobility and diminished quality of life^1,2^. The mechanisms underlying aging are multifaceted and intricate, and numerous theories have been proposed to elucidate the molecular regulation of aging across all systems in an organism. Cellular homeostasis is often disrupted in senescent cells, leading to the increased oxidative stress and impaired cytoprotection^3–5^. Growing evidence suggests that the gut microbiota is affected by aging. Dysbiosis of the gut microbiota causes intestinal metabolite abnormalities and allows harmful substances to enter the circulation. Strategies to reshape the gut microbiome can ameliorate some of the age-related effects of host physiology^6,7^.

*Acer truncatum*, a native tree species that thrives in northern China, is a versatile producer of seed oil^8,9^. It is customary to drink *A. truncatum* leaves as an herbal tea in northern China and it is used as a medicinal herb in Inner Mongolia^10,11^. Recently, the Chinese government has accepted the application of *A. truncatum* leaves as a new food raw material, and it will soon enter the market as a new food raw material. *A. truncatum* leaves contains a variety of active ingredients, such as chlorogenic acid, quercetin, myricetin, Succinic acid, Glycyrrhizin and Azelaic acid^12^. Chlorogenic acid is a widely distributed natural compound found in various plant and is one of the most potent phenolic acid compounds^13^. Chlorogenic acid regulates lipid and glucose metabolism, has immunomodulatory and antioxidant effects, and is widely used in the food, chemical, and pharmaceutical healthcare industries^14^. Quercetin is a powerful antioxidant and is thought to have protective anti-aging properties^15^. Several studies have demonstrated that quercetin possesses antioxidant, anti-diabetic properties and is a crucial factor in the prevention of age-related ailments^16^. Myricetin is a flavonoid that is mainly found in vegetables, fruits, and red wine and can effectively protect against osteoporosis, inflammation, and other diseases^17^. Myricetin is regarded as a potentially valuable adjunct to the prospective advancement of neurodegeneration in the post-ischemic brain^18^. Succinic acid serves as a crucial metabolic intermediate in the tricarboxylic acid (TCA) cycle within host cells and is extensively utilized across various industries, including food, pharmaceuticals, surfactants, and biodegradable plastics^19,20^. Glycyrrhizin has been shown to significantly enhance the therapeutic effects of daphnetin, the principal component derived from Daphne cortex, thereby improving its efficacy in treating rheumatoid arthritis (RA)^21^. Azelaic acid is a complex molecule exhibiting diverse biological activities and possesses numerous pharmacological applications in dermatology^22,23^.

Adhesion of gut microbes, which are not absorbed by the body as nutrients, that they can be beneficial and harmful. These substances enter the blood and modulate gene expression, thereby affecting immune and metabolic processes^24^. Owing to its short life cycle and robust fertility, *D. melanogaster* is frequently used as a model in anti-aging research^25^. Its gut exhibits physiological and metabolic similarities to that of mammals. The simplicity of its microbiome presents an opportunity to investigate the relationship between gut microbiome and metabolome after the administration of natural products to the host^26–28^. Therefore, a multi-combination approach was used to conduct the investigation. The application of 16S rRNA gene sequence detection facilitated the identification and analysis of gut microorganisms, while non-targeted metabolomics was used to evaluate the effect of AAL on *D. melanogaster* and identify essential metabolites. This approach proved to be an effective tool for comprehensively studying alterations in metabolites within the host organism.

The present study aimed to demonstrate the anti-aging efficacy of AAL and elucidate its mode of action and potential active ingredients. *D. melanogaster* was used to confirm the in vivo efficacy of AAL. Considering the complex composition and synergistic effects of plant extracts, we determined the chlorogenic acid, neochlorogenic acid, quercetin, myricetin and esculin content in AAL by UPLC-QTOF-MS/MS, and applied the combined multi-omics analysis to explore the underlying mechanism of AAL effects. This study provides a novel perspective on the effects of AAL on aging retardation.

## Materials and methods

### Preparation of AAL

AAL was extracted from the leaves of *A. truncatum* in August. *A. truncatum* is cultivated on the campus of Northwest A&F University, the plant specimens are stored in the Forest Herbaria, College of Forestry, Northwest A & F University (No. 0456993). The plant was taxonomically identified by professor Zhao Shanting from Northwest A&F University. The leaves of *A. truncatum* were cleaned, dried, and then crushed. Subsequently, 20 g of the powdered leaves was accurately weighed and placed into a 500 mL distillation flask. To this, 200 mL of distilled water was added, and the mixture was allowed to soak for 30 min. The mixture was subjected to thermal reflux extraction at 100 °C for 30 min. Following filtration through gauze, the extraction solution was decanted, and the thermal reflux extraction was repeated by adding 200 mL of distilled water to the filter residue, with a total of three extractions performed. The liquid obtained from three extractions was concentrated under reduced pressure *using* a rotary evaporator, maintained at a vapor pressure of 1.75 kPa and a temperature of 20 °C. The concentrated extract was transferred to a 50 mL tube and freeze-dried using a vacuum freeze-dryer set to − 40 °C and a vacuum pressure of 20 Pa. The freeze-dried product was then stored in a dry, cool environment.

### Component analysis of AAL

The analysis of AAL components was performed in two stages. First, the UPLC-MS/MS profile of AAL was obtained and analyzed by Mabwell (Shanghai) Biotech Co., Ltd. (Detailed test methods are provided in the Supplementary Materials). Subsequently, compounds with the highest relative abundance were selected. The concentrations of chlorogenic acid, neochlorogenic acid, quercetin, myricetin, and esculin in AAL were quantified using UPLC-QTOF-MS/MS with suitable internal standards. After thawing the samples from − 80 °C, 0.25 g of sample was mixed and transferred into a 50 mL centrifuge tube. To this, 50 mL of distilled water was added, followed by vortexing for 10 min. The mixture was then centrifuged at 12,000 rpm and 4 °C for 10 min. The supernatant was filtered through a microporous filter membrane (0.45 μm) and stored in a sample flask for UPLC-QTOF-MS/MS analysis. Detection was conducted using a Triple Quad 5500 + QTRAP Ready (SCIEX, AB), the column model was Kinetex 1.7 μm C18 10A, LC Column100 X 2. 1 mm.The analytical conditions were as follows: the mobile phase consisted of 0.5% acetic acid in water for phase A and acetonitrile for phase B. The injection volume was 5 μL, flow rate was 0.2 mL/min, and column temperature was 30 °C. The elution gradient was as follows: 0–3.0 min, 0 − 2% B; 3.0–6.0 min, 6–15% B; 7.0–11.0 min, 15–30% B; 11.0–13.0 min, 30% B; 13.0–14.0 min, 30–95% B; 14.0–17.0 min, 95% B; and 17.0–18.0 min, 30% B. The SCIEX Triple Quad 5500 + LC–MS/MS system equipped with Linear Ion Trap (LIT) function and positive and negative ion operation mode was used. ESI source operating parameters are as follows: ion source, turbo spray; The source temperature was 500 °C; Ion spray voltage (IS) 5500 V (positive ion mode)/− 4500 V (negative ion mode); The ion source gas I(GSI), II(GSII), and curtain gas (CUR) were set at 50, 50, and 35.0 psi, respectively. Collision-activated dissociation (CAD) was medium. Instrument tuning and mass calibration were performed with 10 and 100 μmol/L polypropylene glycol solutions in QQQ and LIT modes, respectively. The standard curves were constructed for the quantification of chlorogenic acid, neochlorogenic acid, myricetin, quercetin, and esculin in AAL (Detailed test methods are provided in the Supplementary Materials).

### *D. melanogaster* stocks and cultures

All flies used in the experiments were wild-type *Berlin-K (8522)* genotype *D. melanogaster* (Northwest A&F University, Yangling, Shaanxi, China). All stocks were maintained in a standard cornmeal medium for *D. melanogaster* in an incubator at 25 °C and 40–50% humidity. To determine the appropriate dose for *D. melanogaster*, gradient concentrations of AAL were added to record survival days. AAL was incorporated into the corn-agar medium at final concentrations of 0.5%, 1%, 2%, and 4% (medium composition is detailed in the Supplementary Materials). Standards for the main active ingredients (chlorogenic acid, neochlorogenic acid, quercetin, and myricetin) were selected, and a mixed standard medium (MS) was prepared based on the quantitative results and optimal concentrations to investigate the functions of these active ingredients. Female and male flies were maintained on corn-agar medium or medium containing AAL, which was replaced every 3–4 days throughout their lifespan. The flies were kept on a 12-h light/dark cycle. Lifetime analysis was conducted using the OASIS 2 software (https://sbi.postech.ac.kr/oasis2/). After determining the optimal concentration, half of the male and female flies were maintained on basal medium, while the other half were exposed to AAL medium at the optimal concentration. The young group consisted of 7-day-old flies fed on a basic diet, the aged group comprised 30-day-old flies also fed on a basic diet, and the AAL group included 30-day-old flies maintained on the optimal concentration of AAL. Within each group, varying numbers of flies were randomly selected for climbing efficiency, cold stress resistance assays, the “Smurf” assay, gut microbiota analysis, and metabolomics analysis.

### Climbing efficiency(Negative geotaxis assay)

The motor ability of flies was determined by a negative geotaxis test. On the day of assay, the flies were transferred to a glass tube (22 cm height, 1.0 cm diameter) under CO_2_ anesthesia. After a 15-min recovery period, the tube was placed vertically for measurement. During the experiment, all flies were shaken to the bottom of the tube. The climbing index was positioned 8 cm from the bottom, and the number of flies that crossed this index within 12 s was recorded. Each treatment included 5 tubes, with 20 flies (half male and half female) per tube. Each tube was tested 5 times, with a 15-min rest between tests. The proportion of flies crossing the climbing index was calculated, excluding the maximum and minimum values for each tube. N indicates the number of trials in each group.

### Cold stress resistance assays

The effect of AAL on the stress resistance of *D. melanogaster* was investigated by subjecting 20 flies (equally divided between males and females) to CO_2_ anesthesia, followed by their placement in an empty culture tube at 0 °C for 2 h. Subsequently, the flies were transferred to room temperature, and the proportion of flies that recovered after 30 min was recorded. There were 7 tubes of flies in each group. The maximum and minimum values in each group were excluded, and N represents the number of tubes in each group.

### “Smurf” assay

To investigate the effect of AAL on intestinal integrity, flies were reared on medium supplemented with or without AAL until the day of “Smurf” test. The staining medium consisted of standard medium prepared with blue dye. The flies were placed in the staining medium overnight (8 h) following 8 h of starvation. A fly was classified as a Smurf when the blue dye was observed outside the digestive tract (Fig. S1). Eight tubes were prepared for each treatment, with 20 flies (half male and half female) per tube. After the experiment, the flies were anesthetized with CO2 and examined under a stereoscope to determine the proportion of Smurfs.

### Gut microbiota analysis

*D. melanogaster* was anesthetized and placed in a glass dish containing normal saline. Intestinal tissues were collected under a stereomicroscope, with tissues from 50 flies pooled into one sample, and six samples were collected from each group. Gut microbiota sequencing was performed in four steps: (a) genomic DNA extraction and purity assessment, (b) quantification and identification of PCR products, (c) mixing and purification of PCR products, and (d) library preparation and sequencing. Data analysis of the gut microbiota was conducted using the following seven steps: (a) double-end joint assembly and quality control, (b) OTU clustering and species annotation, (c) OTU statistics, (d) α diversity analysis, (e) β diversity analysis, (f) species difference analysis, and (g) network and predictive analytics (detailed procedures are provided in the Supplementary Materials).

### Metabolomics analysis

*D. melanogaster* was anesthetized and placed in an anesthesia tray, and the abdominal tissue was dissected and isolated under a stereoscopic microscope. Thirty *D. melanogaster* flies were collected as one sample, and six samples were collected from each group. The metabolomics analysis was divided into three steps: (a) metabolite extraction, (b) onboard testing, and (c) data processing. Metabolomics data analysis was performed in five steps: (a) data preprocessing, (b) Principal Component Analysis (PCA), (c) differential metabolite screening, (d) differential metabolite analysis, and (e) statistical validation. Detailed procedures are provided in the supplementary materials.

### Statistical analysis

Data are expressed as mean ± SEM. Normality testing (Shapiro–Wilk test) was performed using SPSS 25.0 software, with subsequent selection of either one-way ANOVA (for normally distributed data) or the Mann–Whitney U test (for non-parametric data). Gender-specific analyses were exclusively conducted for lifespan studies, where male and female fruit flies were counted separately. All other datasets were analyzed as combined groups without gender stratification. Statistical significance is denoted by *P < 0.05 and **P < 0.01.

## Results

### Quantification of the main ingredients of AAL

**Fig. 1:**
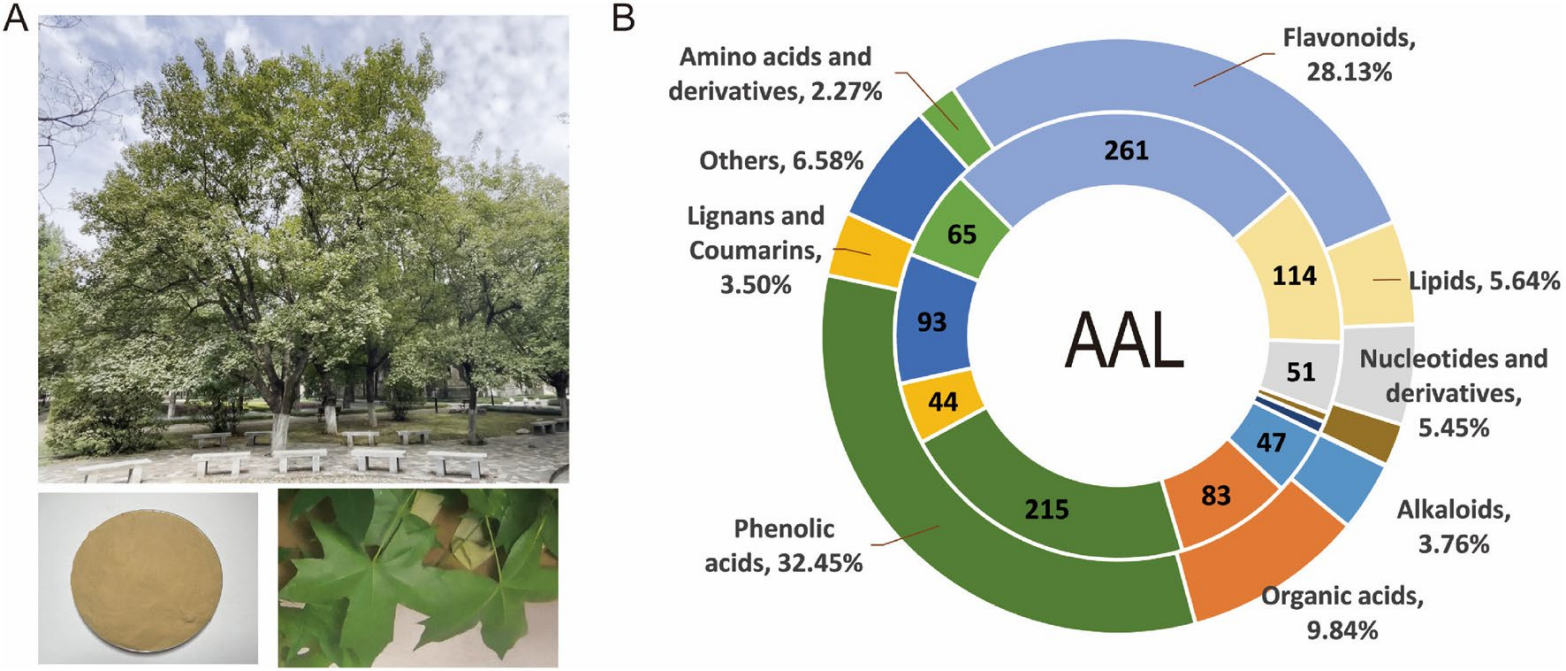
Preparation (**A**) and UPLC-MS/MS analysis (**B**) of aqueous extract of *Acer truncatum* leaves.

*Acer truncatum* is a special deciduous tree in northern China. The aqueous extract of *Acer truncatum* leaves is light yellow powder (Fig. 1A). The present study employed a UPLC-MS/MS approach to initially analyze AAL, resulting in the detection of a total of 226 compounds (Fig. 1B). These compounds could be divided into 11 primary classes, and phenolic acids (32.45%) and flavonoids (28.13%) were the most abundant compounds. The most abundant phenolic acids were Chlorogenic acid, 3-*O*-p-Coumaroylquinic acid and Neochlorogenic acid. The most abundant flavonoids were Quercitrin and Myricetin. In the Lignans and Coumarins, the content of Esculin is very high (Table S1).

**Fig. 2:**
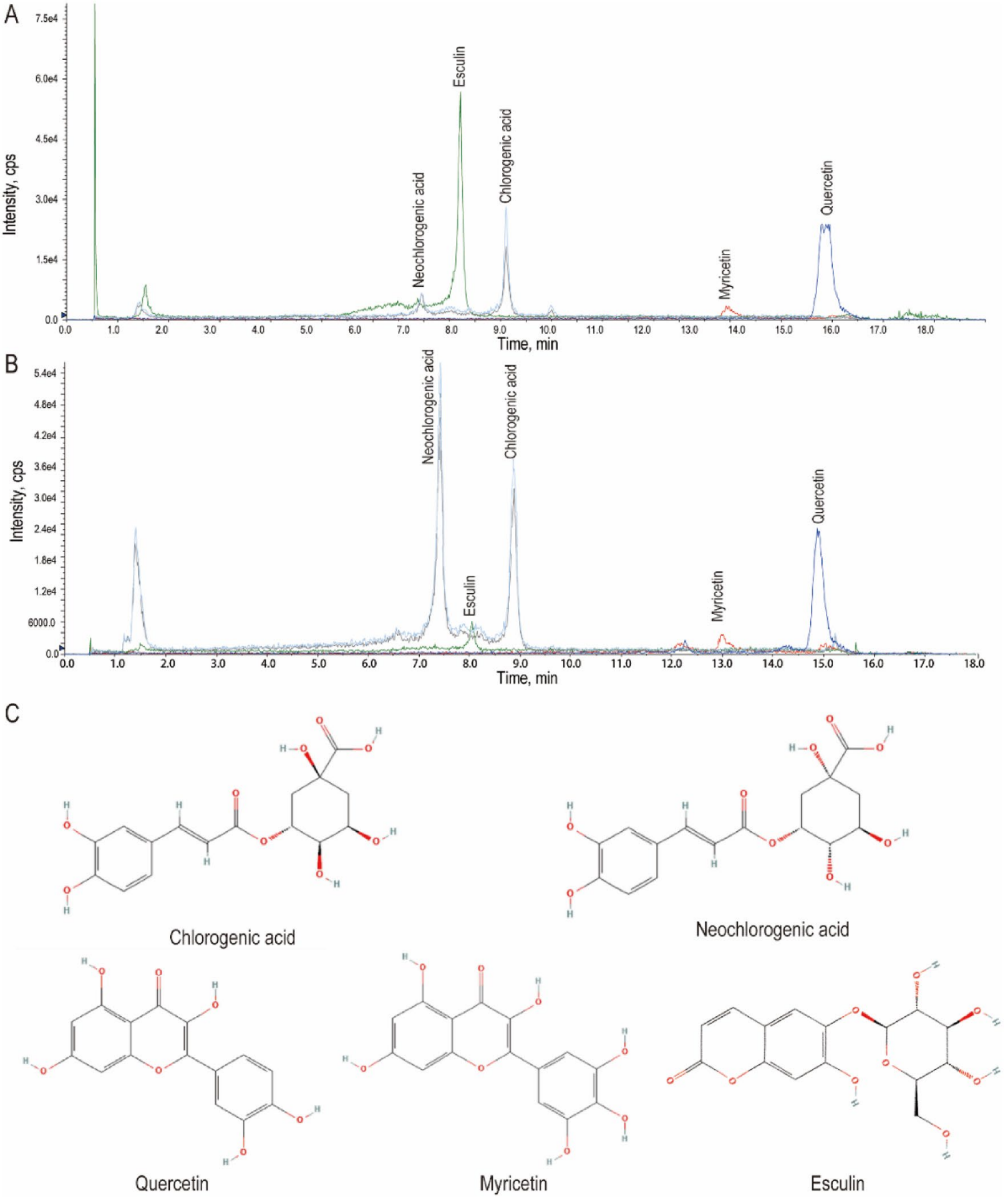
UPLC-QTOF-MS/MS Chromatograms of (**A**) standards of main components and (**B**) AAL, as well as (**C**) their chemical structures.

Figure 2 displays the UPLC-QTOF-MS/MS chromatograms of the main ingredients of AAL and their authentic standards. The calculation is based on the peak area and the concentration standard curve of each compound, respectively (Fig. S3). The contents of the main ingredients in AAL were quantified as followed: chlorogenic acid, 2.902 mg/g; neochlorogenic acid, 17.94 mg/g; quercetin, 1.197 mg/g; myricetin, 3.44 mg/g; and esculin, 0.326 mg/g.

### AAL extends the life-span of *D. melanogaster*

**Fig. 3:**
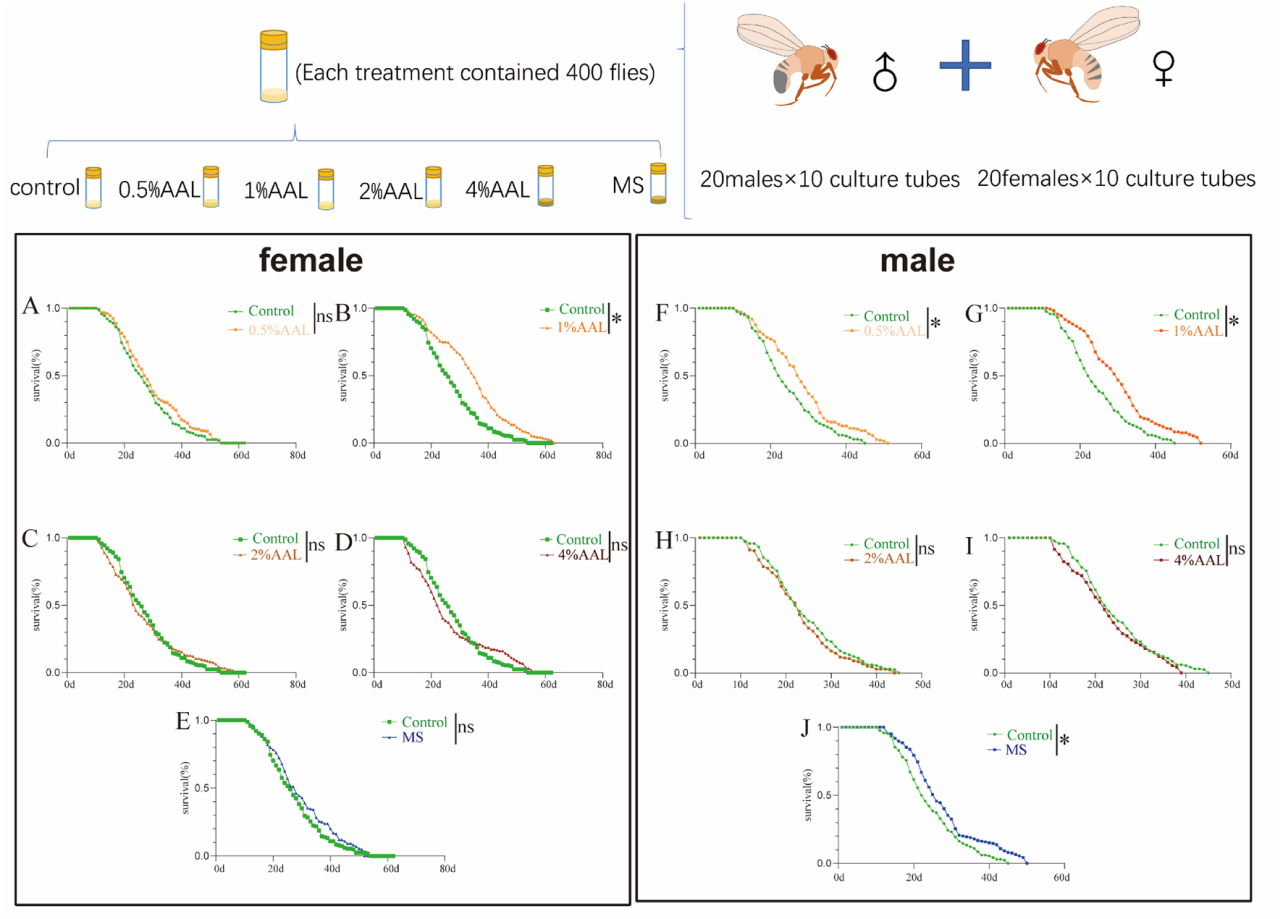
AAL extends the life-span of *D. melanogaster*. Survival of female flies (*n* = 200) after supplementation with 0.5%, 1%, 2%, and 4% AAL feed supplementation (**A**–**D**)*.* Survival of male flies (*n* = 200) after supplementation with 0.5%, 1%, 2%, and 4% AAL feed supplementation (**F**–**I**). Survival of female flies (*n* = 200) and male flies (*n* = 200) after supplementation with mixed standard feed supplementation (**E**, **J**). *P*-values were determined Mantel-Cox test, *P < 0.05.

Phenolic acids and flavonoids have anti-inflammatory and anti-aging effects. We used *Drosophila* model to explore the effects of AAL. We first investigated the effects of dietary supplementation with AAL on the survival ability of *D. melanogaster* by adding four different concentrations of AAL (0.5%, 1%, 2%, and 4%) to the culture medium for *D. melanogaster*. As shown in Fig. 3A–D, concentrations of 1% significantly prolonged the lifespan of female *D. melanogaster*. As shown in Fig. 3F–J, concentrations of 0.5% and 1% significantly prolonged the lifespan of male *D. melanogaster*, among which 1% concentration dramatically extended the lifespan of male *D. melanogaster*. This indicates that supplementation in medium with 1% AAL has noticed anti-aging effects. So, we used 1% AAL as the optimal supplemental concentration for further experiments, in which the different behavior tests were performed. The mixed standard substance (chlorogenic acid, neochlorogenic acid, quercetin and myricetin) with higher content in AAL was mixed in proportion to the same content as 1%AAL, and feeding significantly extended the lifespan of male flies (Fig. 3J), but not for female flies (Fig. 3E).

**Fig. 4:**
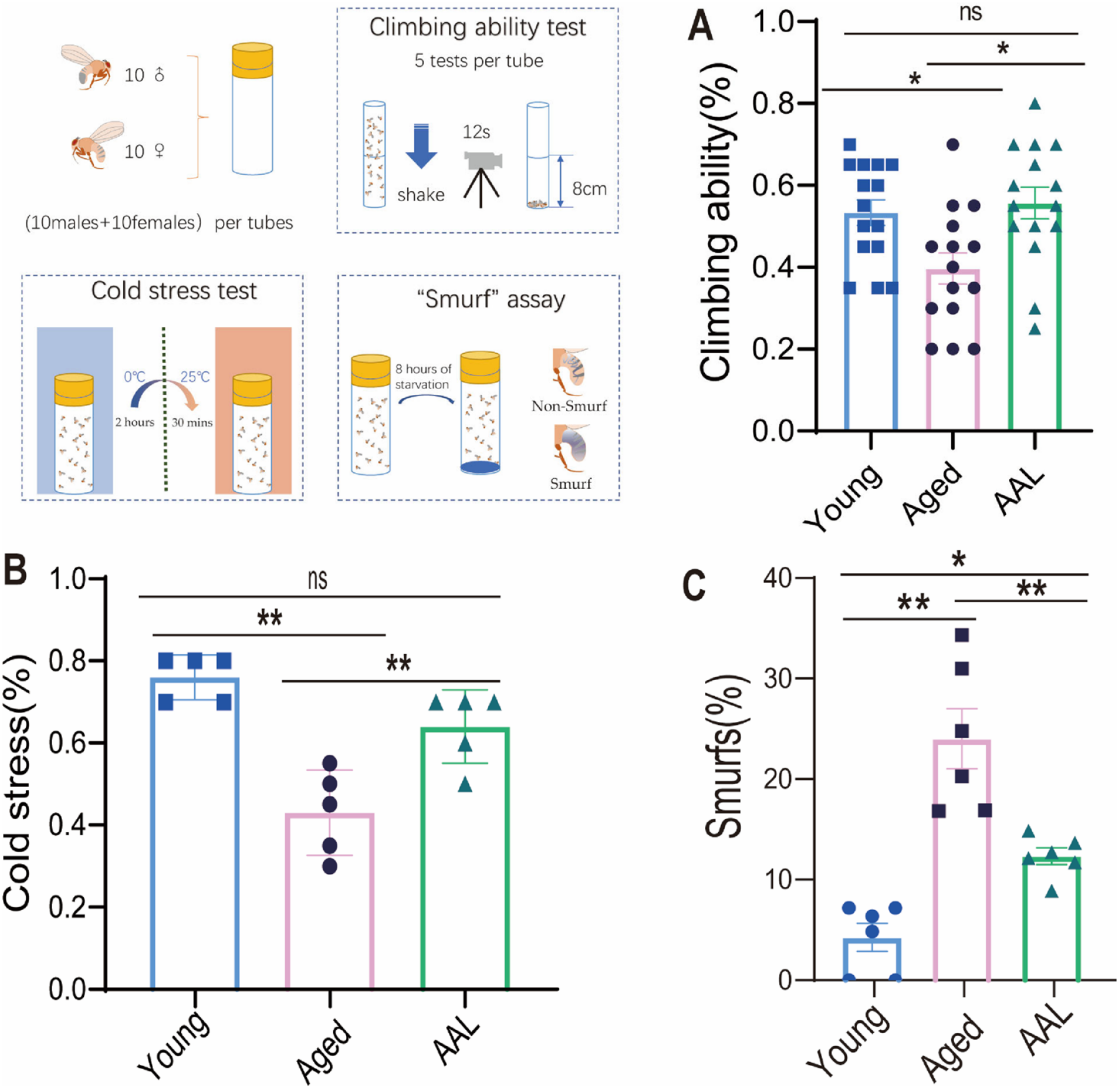
AAL consumption improved age-related behavioral abnormalities and intestinal permeability of *D. melanogaster*. (**A**) Climbing ability test, recording the proportion of *D. melanogaster* climbing over 8 cm within 12 s (*n* = 15 times). (**B**)Cold stress test experiment: *D. melanogaster* was placed at 0 °C for 2 h and then moved out to room temperature. Record the proportion of *D. melanogaster* awakening after 30 min (*n* = 5 tubes). (**C**) Percentage of Smurfs in the young, aged and AAL groups (*n* = 6 tubes). Data are expressed as mean ± SEM of each group. Shapiro–Wilk test (*P* > 0.05), *P*-values were determined using one-way ANOVA, followed by Tukey’s multiple comparison test; *P < 0.05, **P < 0.01.

Aging is often accompanied by the decline of exercise capacity, stress resistance and intestinal sealing damage. We used *D. melanogaster* model to carry out experiments on exercise, stress resistance and intestinal sealing. To investigate the effects of AAL on physical ability of the aged flies, climbing test was performed. Our results showed that the climbing ability of the aged group (30-day old flies, 30d) was significantly lower than that of the young group, while supplementation with AAL enhanced the climbing ability of the aged flies, which suggests that AAL makes the aged flies maintain good physical ability (Fig. 4A). The ability to resist external stress is a sign of an organism’s health and declines with age. The proportion of recovery after cold stress in the aged group was significantly lower than that in the young group, and the proportion of recovery in the AAL group was also significantly higher than that in the aged group, indicating that AAL improves the stress resistance of aged flies (Fig. 4B). Smurf assay showed that compared to the young group more Smurfs were found in the aged group, indicating that the intestinal integrity of the aged flies was disrupted. However, in AAL-treated *D. melanogaster*, fewer ‘Smurfs’ were found than in the aged group, confirming that AAL preserves the intestinal integrity of aged flies (Fig. 4C). These data suggest that AAL diets can improve health and regulate the tissue homeostasis associated with aging.

### AAL improves the gut microbiota of aging D. melanogaster

**Fig. 5:**
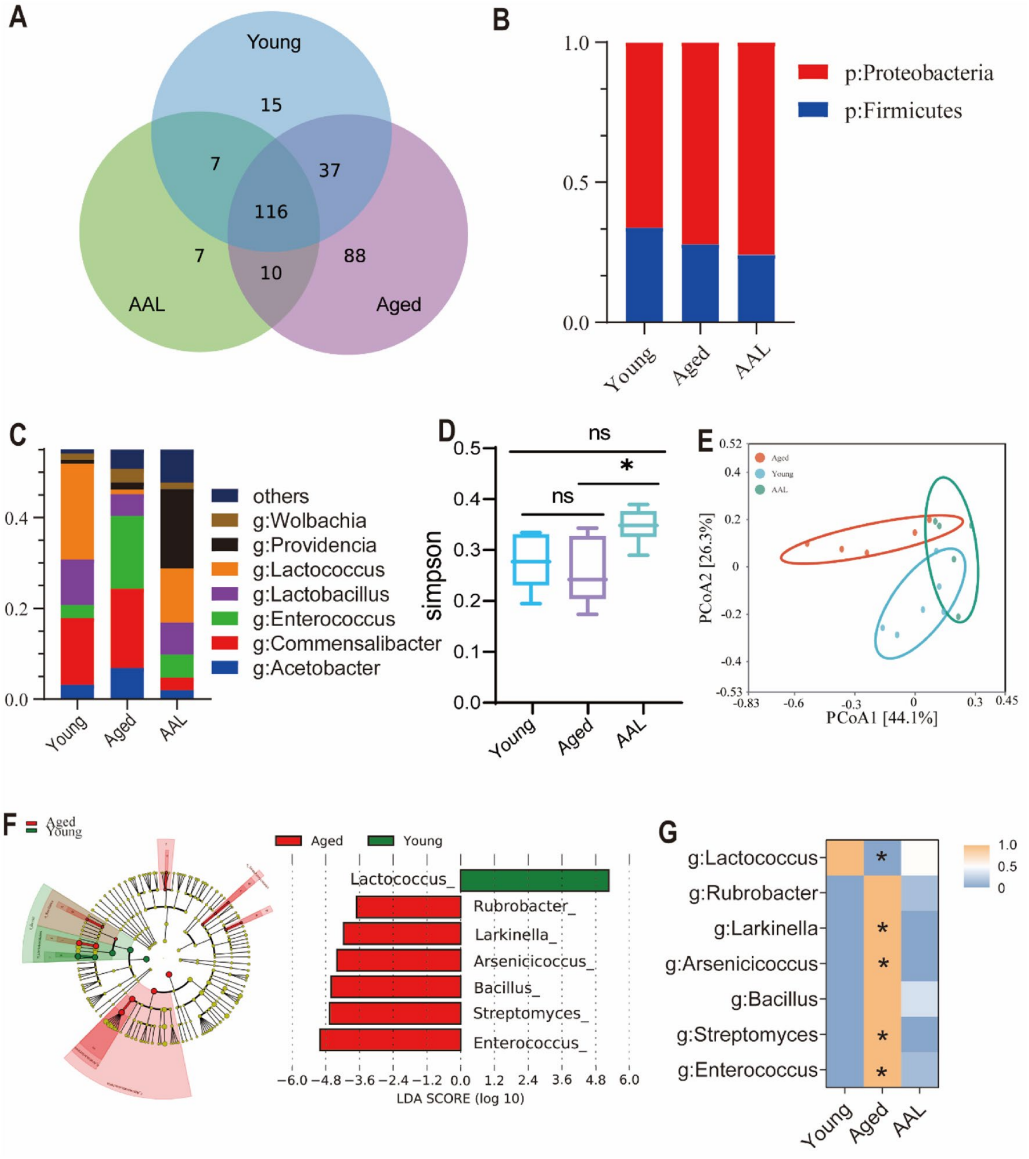
Abundance structure and diversity of microbial communities. (**A**) Venn diagram of OTUS. (**B**) Abundance structure at the phylum level. (**C**) Abundance structure at the genus level. (D) α-diversity (Simpson index). The horizontal line in the box plot represents the mean and the error bars represent the standard deviation. Shapiro–Wilk test (*P * > 0.05), *P*-values were determined using one-way ANOVA; *P < 0.05, **P < 0.01. (**E**) Principal coordinate analysis (PCoA) based on chord distance. (**F**) Evolutionary tree diagram of LEfSe; circles radiating from the inside out represent taxonomic levels from the phylum to the genus, with organisms without significant differences marked in yellow. Biomarkers colored by group, with green, blue, and red indicating the microbial groups that played an important role in the aged, young, and AAL groups, respectively. Histogram of LEfSe parameters: The histogram of the distribution of LDA values shows organisms whose gene abundance differs significantly across populations and the length of the histogram indicates the degree of influence of different organisms (LDA Score). (**G**) Heat map of genus-level abundance. Shapiro–Wilk test (*P* < 0.05), Kruskal–Wallis H test (*P* < 0.05), The ‌Mann-Whitney U method was used between the AAL group and the aged group, as well as between the AAL group and the young group. *P < 0.05.

Although five substances in AAL are known to have anti-inflammatory and anti-aging effects, their mechanisms of action remain unclear. Gut microbiota is closely related to body health, and the effect of AAL is closely related to gut microbiota. We studied the gut microbiota of *D. melanogaster*. Overall, 282 OTUs were detected in the three groups, with 88, 15, and 7 special OTUs in the aged, young, and AAL groups, respectively (Fig. 5A). At the phylum level, the sequenced reads from the samples (content > 0.01%) were assigned to 12 phyla. *Proteobacteria* and *Firmicutes* were the two most abundant phyla in all *D. melanogaster* samples (Fig. 5B). *Proteobacteria* and *Firmicutes* were the major phyla of dominant bacteria in the gut of *D. melanogaster*, accounting for majority of the total bacterial community. Among the three groups, *Proteobacteria* was the most abundant phylum in the AAL group, and *Firmicutes* was the most abundant phylum in the young group. At the genus level, 19 genera were annotated in all the samples, with the top-ranking genera being *Acetobacter*, *Acinetobacter*, *Commensalibacter*, *Enterococcus*, *Lactobacillus*, *Lactococcus*, *Providencia*, *Pseudomonas*, and *Wolbachia* (Fig. 5C). The genera represented in each group were diverse, with *Enterococcus* being the most abundant in the aged group (16.04%), while *Lactococcus* was most abundant in the young group (21.12%) and *Pseudomonas* was the most unusual genus in the AAL group (17.55%) (Fig. S4). Although only a few genera were shared by all species, the 20 most abundant genera represented a significant proportion (98%) of the microbial community in *D. melanogaster*.

To distinguish the difference in the gut microbes of *D. melanogaster*, diversity indices of the microbial communities (α- and β-diversity) were also assessed between the different groups. α-diversity indicated no significant difference in community abundance among the three groups (Supplementary), and the Simpson index suggested that the diversity of bacterial genera was significantly higher in the AAL group than that in the aged group (Fig. 5D). In addition, the results of the principal coordination analysis (PCoA) based on Bray–Curtis distances revealed different natures of bacterial communities in each group (Fig. 5E). LEfSe analysis is a method for finding high-dimensional biosignatures and revealing genomic features using an algorithm that emphasizes statistical significance and biological relevance (Fig. 5F). Age is a crucial determinant of the gut microbiota structure in *D. melanogaster*, with a marked difference in microbiota abundance between aged and young groups. We found 7 bacterial genera that are related to age at the genus level. Respectively, one of the biologically significant microorganisms in the young group belonged to the *Firmicutes* phylum, with the dominant species being *Lactococcus*. In the aged group significant microorganisms belong to *Proteobacteria* and *Actinobacteria* phyla, with the dominant species being *Enterococcus*, *Arsenicicoccus*, *Streptomyces*, *Larkinella, Bacillus* and *Rubrobacter*. The utilization of a genus-level heat map facilitated the visual representation of variations in microbial abundance among the aged, young, and AAL groups (Fig. 5G). Specifically, in AAL group the Lactococcus abundance significant increased and the Enterococcus abundance significant decreased compared to those in the aged group. These results suggested that AAL could reshape the structure of gut microbiota and adjust the abundance of gut microbiota with age, which showed a similar trend to the young group.

### AAL alters the metabolome of aging D. melanogaster

**Fig. 6:**
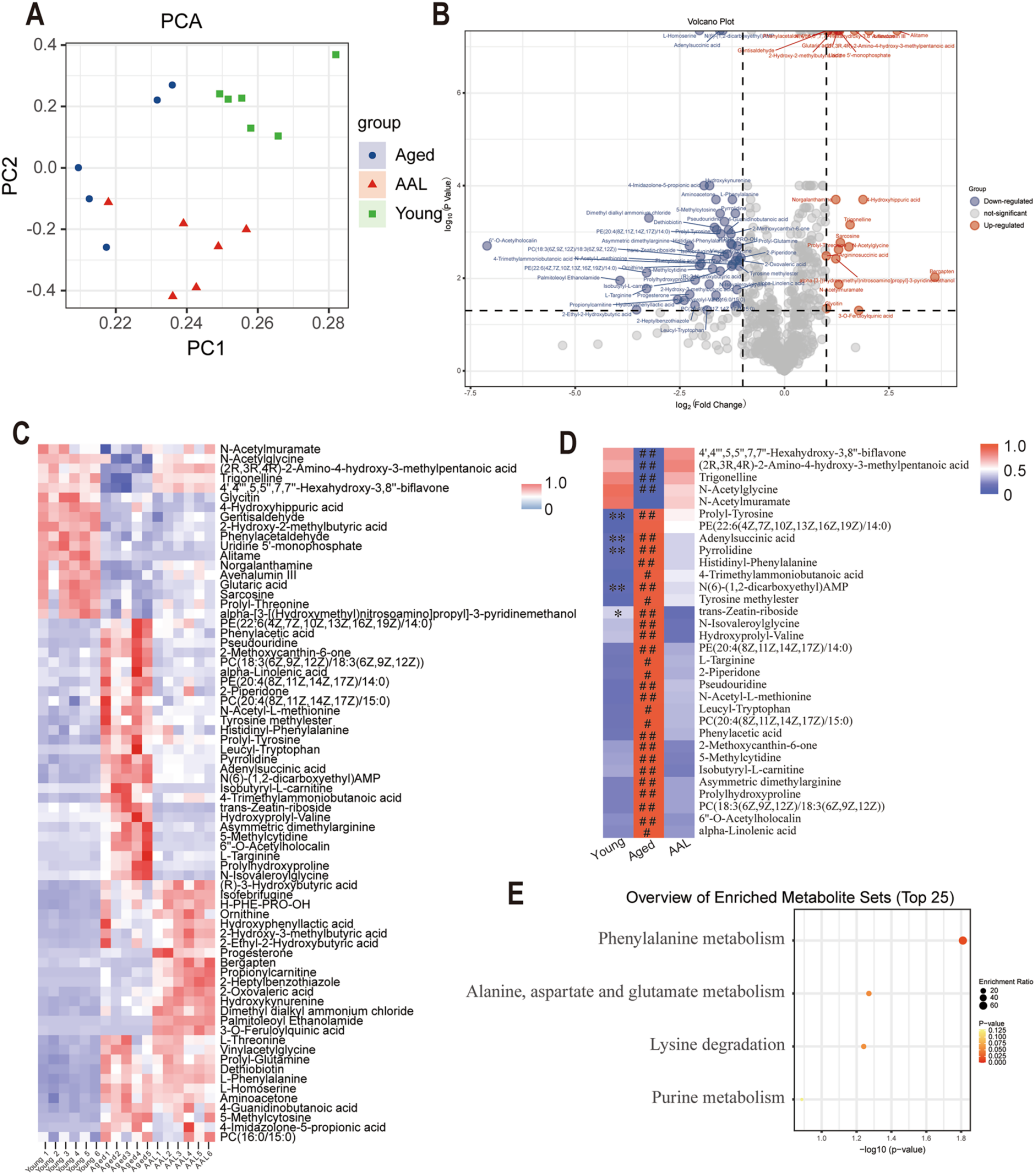
Untargeted metabolomics analysis. (**A**) 2-D principal component analysis (PCA) score plot demonstrating statistical clustering of metabolites in response to aged (green), young (blue), and AAL (red) groups. (**B**)Volcano plot of metabolites with a fold-change threshold (x) of 1.0 and t-test threshold (y) of 0.1. The red circles and the purple circles represent features above the threshold. The farther the position from (0,0), the more significant the metabolite. (**C**) Heat map of the metabolites between the aged and young groups, and the abundance of these differential metabolites in the AAL group. The red color represents upregulated metabolites and the blue circles represent downregulated metabolites. (**D**) Heat map of significant metabolites with the same trend of change in the AAL group and the young group. Shapiro–Wilk test (*P* > 0.05), *P*-values were determined using one-way ANOVA. * Indicates the significant difference between the AAL group and the young group, # indicates the significant difference between the AAL group and the aged group, * # *P* < 0.05, ** ## *P* < 0.01. (**E**) Enrichment analysis of significant metabolites.

Gut microbiota is closely related to host homeostasis and affects host function by regulating metabolism. To investigate the major metabolic changes associated with supplementation of AAL, we used a non-target metabolomics approach to quantify primary and secondary metabolites in adult *D. melanogaster* after AAL diet supplementation. As seen from the overlaid Liquid chromatography–mass spectrometry (LC–MS) total ion chromatograms (TICs) of *D. melanogaster* QC samples, each sample exhibited a corresponding unique metabolite profile in both positive and negative ion modes, with a good overlap of TICs for all combined QC samples. Indicating that the instrumental system burst was stable during sample analysis, resulting in good-quality data for *D. melanogaster* metabolomics (Fig. S6). In addition, QC samples from each type of metabolomic data set were generally well clustered in the corresponding PCA score plots, further indicating that the metabolomic data collection was reliable and ready for subsequent data processing and statistical analysis (Supplementary Figure). All *D. melanogaster* samples were pre-processed with internal standard-based normalized data and 678 metabolites were screened out. The annotated metabolites were divided into different classes, such as benzene and substituted derivatives, carboxylic acids, fatty Acyls, glycerophospholipids, hydroxy acids, organooxygen compounds, pteridines, and quinolines. PCA was performed to investigate the separation of metabolites between the aged, young, and AAL groups. Principal component analysis provided an unbiased picture of the metabolic status of all samples and the three groups were well separated in the principal component score plot, with good concordance within the same group (Fig. 6A). The metabolites between the young and aged groups were compared to elucidate age-related differential metabolites. We screened for differential metabolites using *P* < 0.05, and *FC* ≥ 2 or *FC* ≤ *0.5*, and identified 72 differential metabolites (Fig. 6B). Compared with the young group, 52 different metabolites in the aged group were significantly up-regulated and 20 different metabolites were significantly down-regulated (Fig. 6C). We found that these different metabolites showed different trends in the AAL group and the aged group, indicating that AAL changed the metabolic function of the aging flies. We selected 32 metabolites from the AAL group whose change trend was similar to that of the young group, which might be the key metabolites of AAL delaying aging of *D. melanogaster*. Through further comparative analysis (Fig. 6D), we found that 5 metabolites in the young group were significantly lower than those in the AAL group, 26 metabolites in the aged group were significantly higher than those in the AAL group, and 4 metabolites were significantly lower than those in the AAL group. We selected significantly differential metabolites for enrichment analysis, and the differential metabolites were transferred to the *MetaboAnalyst 6.0*. The top 25 metabolic pathways with the most important number of differentially accumulated metabolites were analyzed (Fig. 6E). We found that these metabolites were mainly involved in the following pathways: phenylalanine metabolism, alanine, aspartate and glutamate metabolism, lysine degradation, purine metabolism.

### Integrated omics analysis for potential metabolites and microbes by AAL

Pairwise correlation analysis and correlation coefficients between differential metabolites and microorganisms were calculated using Spearman’s paired correlation analysis. Red indicates a positive correlation and blue indicates a negative correlation. Darker colors indicate greater statistical significance. The symbols * and ** indicate *P*-values with correlation coefficients of less than 0.05 or 0.01, respectively.

**Fig. 7:**
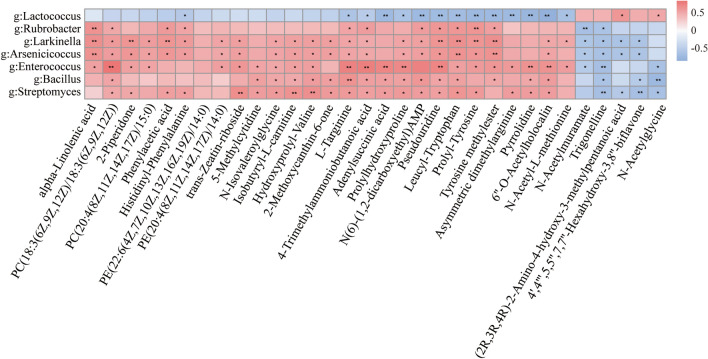
Combined microbiome and metabolomics analysis.

To reveal co-occurrence patterns between gut microbiota and metabolites in *D. melanogaster* after AAL supplementation, we used Spearman’s correlation analysis to determine the strength of association and identified the core microorganisms and metabolites driving this relationship. *Lactococcus* and *Enterococcus* are highly abundant microorganisms in the genus level, and both belong to *Firmicutes*. However, they showed opposite correlations with key compounds (Fig. 7). *Lactococcus* had a significant positive correlation with potential metabolite (2r,3r,4r)-2-amino-4-hydroxy-3-methylpentanoic acid and n-acetylglycine. Among them, n-acetylglycine showed a significant negative correlation with *Enterococcus*. *Lactococcus* has a significant negative correlation with the potential metabolite n-acetyl-l-methionine, 6''-o-acetylholocalin, pyrrolidine, asymmetric dimethylarginine, tyrosine methylester, prolyl-tyrosine, leucyl-tryptophan, pseudouridine, n(6) -(1,2-dicarboxyethyl) amp, prolyl-aspartate, adenylsuccinic acid, 4-trimethylammoniobutanoic acid, l-threonine and histidinyl-phenylalanine. Among them, n-acetyl-l-methionine, 6''-o-acetylholocalin, pyrrolidine, asymmetric dimethylarginine, tyrosine methylester, prolyl-tyrosine, leucyl-tryptophan, pseudouridine, prolyl-aspartate, adenylsuccinic acid, 4-trimethylammoniobutanoic acid, l-threonine and histidinyl-phenylalanine a significant positive correlation with *Enterococcus*. The potential metabolite correlation of other low-abundance bacteria genera was similar to that of *Enterococcus*.

## Discussion

Limited studies have evaluated the anti-inflammatory and anti-aging effects of novel food ingredient AAL. To address this knowledge gap, our study adopted an integrated approach including behavioral assessment, microbiome and metabolomics strategies to elucidate the active ingredients and potential modes of action of AAL in delaying aging in *D. melanogaster*.

*A. truncatum* is a tree species that grows naturally in northern China and is an economic forest species that combines multiple functions such as greening, drought resistance, and oil production^29–31^. In recent years, researchers have placed significant importance on the exploration and advancement of *A. truncatum* resources, with particular attention given to the potential therapeutic properties of seed oil for the treatment of neurological diseases^32,33^. At present, functional diet and food products are used as effective health supplements or botanical drugs in clinical practice to solve various medical problems. In Inner Mongolia, the utilization of *A. truncatum* leaves as an anti-inflammatory agent has been a longstanding tradition among herders. However, the preservation of this relatively obscure traditional medicine is currently at risk due to insufficient scientific investigation^10^. Recently, the Chinese government has accepted the application of the *A. truncatum* leaves as a new food raw material, and it will enter the market as a food raw material in the future, which will greatly promote the development of the *A. truncatum* industry. To investigate the chemical composition and medicinal value of the leaves of *A. truncatum*, we used UPLC-MS/MS to reveal their chemical composition. A total of 226 chemical constituents were detected in the leaves of *A. truncatum*, belonging to 11 major groups. Phenolic, flavonoid, and organic acids accounted for 70.42% of the total content. Chlorogenic acid and Neochlorogenic acid are the most abundant substances in phenolic acids. Chlorogenic acid is ubiquitous in plants and its antimicrobial properties of chlorogenic acid were widely described^34^. Myricitrin and quercitrin were the most abundant flavonoids. Myricitrin has been used in studies on cardiovascular disease and organ damage and has been found to have potent antioxidant and hepatoprotective properties^35^.

Numerous studies have shown that natural products improve human health, mainly through the gut microbiota^36,37^. As many of the organs and tissue networks of *D. melanogaster* function in the similar manner as in most mammals, we sought to use the *D. melanogaster* model to study the effects of natural products on the gut microbiota and metabolism of an organism^38^. Food composition plays a crucial role in determining the longevity of *D. melanogaster*. To investigate this issue, we prepared medium with four different concentrations of AAL and selected 400 newborn *D. melanogaster* with an equal distribution of males and females for each concentration. Our findings indicate that the inclusion of 1% AAL in the medium significantly extended lifespan of female *D. melanogaster*, and the inclusion of 0.5% and 1% AAL in the medium significantly extended lifespan of male *D. melanogaster.* Notably, the 1% AAL group exhibited the most substantial increase in the lifespan, the higher concentrations of AAL had no effect on longevity. The addition of high concentration of AAL can cause odor change and texture hardening of the medium, and the concentration of key components may be an effective scheme to further study the active components of AAL in the future. To investigate the impact of the primary active components in AAL on the lifespan of *D. melanogaster*, we selected the four highest content material standards to create a mixed standard medium and administered it to the flies at a concentration of 1% AAL. The results showed that MS medium could significantly prolong the lifespan of male flies, but had no significant effect on the lifespan of female flies (*P* = 0.4). This suggests that AAL contains other active components that affect the lifespan of *D. melanogaster*. Aging is a natural process that often leads to progressive damage and pathological changes^39^. The climbing ability of *D. melanogaster* in the AAL group was significantly better than that in the aged group, indicating that supplementation of AAL improved the locomotor capacity of the aged *D. melanogaster*. The function of various animal tissues and the body’s ability to self-regulate decreases with age, resulting in reduced resistance to external stresses or adaptations^40^. *D. melanogaster* possesses multiple temperature-sensing systems that regulate the body’s ability to adapt to different temperature changes. Physiological dormancy is the ability of *D. melanogaster* to respond to stress in cold environments^41,42^. Our study showed that after cold stimulation, the proportion of *D. melanogaster* awake in AAL group was significantly higher than that in the aged group. This suggests that dietary supplementation with AAL could improve stress resistance in *D. melanogaster*. The intestinal barrier function is an evolutionarily conserved biomarker and its dysfunction is mainly caused by aging^43^. To investigate the potential restorative effects of AAL on intestinal function, we examined the presence of unabsorbed dye outside the digestive tract of *D. melanogaster* as a measure of compromised intestinal barrier function. Our findings revealed that the aged *D. melanogaster* exhibited the disruption of intestinal integrity indicated by the presence of “Smurfs” flies. However, the proportion of “Smurfs” flies in AAL group was significantly lower than that in the aged group, indicating that dietary supplementation with AAL effectively preserved the intestinal integrity. Our study demonstrated that AAL dietary supplementation not only extended the lifespan of *D. melanogaster* but also improved their physiological functions.

The gut microbiota plays a vital role in maintaining host health, not only by directly affecting the host intestinal environment but also by regulating endocrine, energy metabolism, and immune responses^44^. The establishment and maintenance of gut microbiota are affected by various factors, including diet fluctuation, pathogen invasion, and antibiotic interference, among which dietary habits are most closely related to the composition and function of the microbiota^45,46^. In studying mechanisms and genotypes, multiple dynamics of host-microbiome interactions are often studied using animal models of controlled diets. Compared with mammals, *D. melanogaster* has the advantage of simple gut microbiota^47^. A total of 280 OTUs were annotated by sequencing the 16S rRNA genes from the gut microbes of *D. melanogaster*. The microbial population of the gut microbiota of *D. melanogaster* changes with increasing age and its compositional richness and variability increases^48^. The gut microbiota of *D. melanogaster* is simple and mainly comprises Proteobacteria and Firmicutes. Interestingly, the α-diversity of the gut microbiota of the AAL group was higher than that of the aged group, indicating that the gut microbiota was more diversified due to AAL dietary supplementation. AAL alters the balance of harmful and beneficial bacteria in the intestine and has a weak inhibitory effect on *Pseudomonas* and *Stenotrophomonas*. The abundance of *Lactococcus* decreased significantly with increasing age and almost disappeared in the aged group; however, the abundance in the AAL group still retained a certain amount (*P* = 0.0876). *Lactococcus* is a common probiotic in the intestinal tract of humans and animals and is closely related to the prevention and treatment of gastrointestinal diseases. Supplementation of *Lactococcus* improves the structural disturbance of the flora associated with metabolic syndrome^49,50^. Many studies have found that *Enterococcus* have significant changes in many diseases. For example, it was found that the abundance of *Enterococcus* significantly increased in liver fibrosis caused by a high-salt diet in the acute pancreatitis mice, as well as in patients with Crohn’s disease^51,52^. Our study found that *Enterococcus* abundance significantly increased with age and remarkably decreased after AAL dietary intervention. Based on our results, we concluded that AAL prolongs the lifespan of *D. melanogaster* by improving intestinal microbial homeostasis.

Changes in gut microbiota may lead to metabolic changes in the host through microbial metabolites. We explored age-related metabolomic differences in *Drosophila melanogaster* and found 72 potential metabolites that changed significantly with age, which may be related to the aging degradation of tissue function. We screened the potential metabolites of AAL in delaying *Drosophila* aging, and 32 potential differences were found. Metabolite enrichment analysis showed that these potential metabolites mainly affected metabolic pathways involved in Phenylalanine metabolism, Alanine, aspartate and glutamate metabolism, Lysine degradation, Purine metabolism. Phenylalanine is one of the essential amino acids in human body. Tyrosine can be formed by the action of hepatic phenylalanine hydroxylase^53^. As individuals age, the body’s requirement for phenylalanine to synthesize proteins gradually diminishes, resulting in an accumulation of phenylalanine^54^. This accumulated phenylalanine undergoes transamination to yield phenylpyruvate, which is subsequently converted into various derivatives such as phenylacetic acid^55^. In the present study, phenylacetic acid was significantly increased in the aged group. Alanine and aspartate are non-essential amino acids that can be produced by microorganisms with various enzymes. They can be used as raw materials or starting materials for industrial production of food, cosmetics, pharmaceuticals and other products.

Diet is closely related to the changes in metabolites of *D. melanogaster*, which is also the main reason for the changes in gut microbiota^56,57^. We explored the correlation between the latent microbiota and metabolites in *D. melanogaster* after AAL ingestion. Correlation analysis showed that among the potential microorganisms and metabolites of *D. melanogaster*, The potential metabolites trigonelline were positively correlated with *Lactococcus* and significantly negatively correlated with *Enterococcus*. Trigonelline is a natural alkaloid found in plants and animals, including in humans^58^. Trigonelline is a highly effective antibacterial agent and has a good inhibitory effect on a variety of refractory pathogenic bacteria^59^. *Lactococcus* has a significant negative correlation with the potential metabolite n-acetyl-l-methionine, 6''-*O*-acetylholocalin, pyrrolidine, asymmetric dimethylarginine, tyrosine methylester, prolyl-tyrosine, leucyl-tryptophan, pseudouridine, n (6) -(1,2-dicarboxyethyl) amp, prolyl-aspartate, adenylsuccinic acid, 4-trimethylammoniobutanoic acid, l-threonine and histidinyl-phenylalanine. Supplementation of l-threonine could prevent age-related iron concentration. Ferritin level was positively correlated with aging and l-threonine was up-regulated in aged animals^60^. The elevated level of asymmetric dimethylarginine is a marker of chronic renal failure and vascular endothelial injury. Reducing the level of asymmetric dimethylarginine is an important scheme to inhibit the progression of cardiovascular diseases^61^. *Lactococcus and Enterococcus* showed opposite correlations with potential metabolites, indicating that AAL can improve the structure of gut microbiota in *D. melanogaster* and regulate the concentration of metabolites, thereby prolonging the lifespan of the *D. melanogaster*.

## Conclusions

In summary, the present study revealed the characteristics of gut microbiota and metabolome in *Drosophila* in which AAL delayed aging, and analyzed the interaction between them in related studies. AAL increases the diversity of *Drosophila* gut microbes and the abundance of beneficial bacteria, and reduces the production of harmful metabolites by regulating metabolite pathways. The results of our study provide beneficial implications for the anti-inflammatory and anti-aging effects of AAL, which is helpful for the utilization of the resources of the *A. truncatum* leaves and the development of functional food.

## Supplementary Information

Supplementary Information 1.

Supplementary Information 2.

Supplementary Information 3.

## Data Availability

Data is provided within the manuscript or supplementary information files.
