# Supplementary material for: Aqueous extract of Acer truncatum leaves retards Drosophila melanogaster senescence by regulating amino acid metabolism and gut microbiota
Source: Sci Rep. 2025 Oct 2;15:34409. doi: 10.1038/s41598-025-17390-7 (PMC12491420; doi:10.1038/s41598-025-17390-7)
Supplement: Supplementary file 3 — Supplementary Information 3. [file 41598_2025_17390_MOESM3_ESM.docx]

**Supplementary**

**Methods**

**1.** **Method** **for UPLC-MS/MS profile of AAL**

**1.1 Sample preparation and extraction**

1.1.1 After thawing the samples from the refrigerator at -80℃, mix them with vortex for 10 s.

1.1.2 Take 6mg of the sample after mixing, place them into a centrifuge tube with 50mL volume, immerse the sample with tube into liquid nitrogen. Put the sample into the lyophilizer for freeze-drying after the sample was completely frozen.

1.1.3 After the samples were completely lyophilized, take 50 mg of the sample after mixing, place them into a centrifuge tube with 2mL volume, 1200ul 70% methanol internal standard extract was added. Scroll for 3 min.

1.1.4 Centrifuge (12000 r/min, 4℃) for 10min. The supernatant was filtered with a microporous filter membrane (0.22μm) and stored in a sample flask for LC-MS/MS test.

**1.2 UPLC Conditions**

The sample extracts were analyzed using an UPLC-ESI-MS/MS system (UPLC, SHIMADZU Nexera X2, https://www.shimadzu.com.cn/; MS, Applied Biosystems 4500 Q TRAP, https://www.thermofisher.cn/cn/zh/home/brands/applied-biosystems.html). The analytical conditions were as follows, UPLC: column, Agilent SB-C18 (1.8 µm, 2.1 mm * 100 mm); The mobile phase was consisted of solvent A, pure water with 0.1% formic acid, and solvent B, acetonitrile with 0.1% formic acid. Sample measurements were performed with a gradient program that employed the starting conditions of 95% A, 5% B. Within 9 min, a linear gradient to 5% A, 95% B was programmed, and a composition of 5% A, 95% B was kept for 1 min. Subsequently, a composition of 95% A, 5.0% B was adjusted within 1.1 min and kept for 2.9 min. The flow velocity was set as 0.35 mL per minute; The column oven was set to 40℃; The injection volume was 4 μL. The effluent was alternatively connected to an ESI-triple quadrupole-linear ion trap (QTRAP)-MS.

**1.3 ESI-Q TRAP-MS/MS**

LIT and triple quadrupole (QQQ) scans were acquired on a triple quadrupole-linear ion trap mass spectrometer (Q TRAP), AB4500 Q TRAP UPLC/MS/MS System, equipped with an ESI Turbo Ion-Spray interface. Operating in positive and negative ion mode and controlled by Analyst 1.6.3 software (AB Sciex). The ESI source operation parameters were as follows: ion source, turbo spray; source temperature 550℃; ion spray voltage (IS) 5500 V (positive ion mode)/-4500 V (negative ion mode); ion source gas I (GSI), gas II(GSII), curtain gas (CUR) was set at 50, 60, and 25.0 psi, respectively; the collision-activated dissociation (CAD) was high. Instrument tuning and mass calibration were performed with 10 and 100 μmol/L polypropylene glycol solutions in QQQ and LIT modes, respectively. QQQ scans were acquired as MRM experiments with collision gas (nitrogen) set to medium. DP and CE for individual MRM transitions was done with further DP and CE optimization. A specific set of MRM transitions were monitored for each period according to the metabolites eluted within this period.

**2. Standard preparation and quantitative concentration calculation**

Add 1 mL methanol to the standard vial, mix to prepare a 20 mg/mL standard stock solution. Dilute with methanol to prepare gradient standards of 10 µg/mL, 20 µg/mL, 50 µg/mL, 100 µg/mL, 200 µg/mL, 500 µg/mL, and 1000 µg/mL. After UPLC-QTOF-MS/MS detection, standard curves were constructed, and the R² values for each curve were found to be > 0.98, confirming the validity of the curves. The prepared 5 mg/mL AAL solution was analyzed using UPLC-QTOF-MS/MS to obtain the peak area, which was then used to calculate the relative content based on the standard curves. Therefore, the content of this substance in AAL was calculated as follows:

If the standard curve is: Y= AX+B (A, B are natural numbers)

$$M=\frac{(\frac{Ya-B}{A})\times C}{5 mg/mL\times C}\times1000$$

M, content of components (mg/g); Ya, peak area of AAL at this standard position; C, Injection volume

**3. Medium components**

Corn-agar medium with the following proportions: 720 mL H_2_O, 72 g Corn meal, 48 g Sucrose, 9g yeast powder, 6 g AGAR powder and 2.4 mL propionic acid. The amount of AAL was added in proportion to the total media mass, and part of the corn meal content was replaced by AAL. The calculation formula is as follows:

$$X\%=\frac{M g}{720g H2O +\left( 72-M \right)g Corn meal+Mg AAL+48g Sucrose +9g yeast powder +6 g AGAR powder}\times100\%$$

M, The weight of AAL (g)

**4. Representative photo of *D.melanogaster* "*Smurf*" compared to "*non-Smurf*"**

**
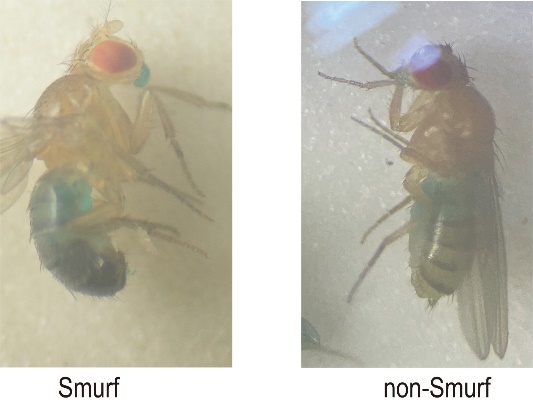
**

**Figure S1**. *D.melanogaster* "*Smurf*" compared to non-Smurf

**5.Gut microbiota analysis**

**5.1 Experimental procedure**

**5.1.1 DNA extraction**

The purity and concentration of genomic DNA were measured using the Thermo NanoDrop One following extraction with the corresponding DNA extraction kits.

**5.1.2 PCR amplification and product electrophoresis detection**

Genomic DNA was used as template for PCR amplification using specific primers with barcode and TaKaRa Premix Taq® Version 2.0(TaKaRa Biotechnology Co.Dalian, China) according to the selection of the sequencing region.

5.1.2.1 Regions corresponding to primers

16SV3-V4

5.1.2.2 PCR reaction system

| Reagent name | Dosage |
| --- | --- |
| 2x Premix Taq | 25 μl |
| Primer-F（10 μM） | 1 μl |
| Primer-R（10 μM） | 1 μl |
| DNA | 50 ng |
| Nuclease-free water | add to 50 ul |

5.1.2.3 PCR reaction conditions

1) 94ºC 5min

2) 30 cycles of

94ºC 30s

52ºC 30s

72ºC 30s

3) 72ºC 10min

4) 4ºC Hold

Each sample was performed in triplicate, and the PCR products from the same sample were pooled, PCR instrument: BioRadS1000 (Bio-Rad Laboratory, CA).

5.1.2.4 Electrophoresis detection of PCR products

Fragment length and concentration of PCR products were examined by 1% agarose gel electrophoresis, and samples with major band lengths within the normal range (16S V3-V4:400-450bp) were available for further experiments.

**5.1.3 Pooling and gel cutting purification**

GeneTools analysis software (Version4.03.05.0, SynGene) was used to compare the concentration of PCR products. The required volume of each sample was calculated according to the principle of equal mass, and the PCR products were mixed.

The PCR mixed products were recovered using the E.Z.N.A.® Gel Extraction Kit (Omega, USA) and the target DNA fragments were recovered by elution with TE buffer.

**5.1.4 Library construction and sequencing**

5.1.4.1 Library construction

Library construction was performed according to the standard procedure of NEBNext® Ultra™ II DNA Library Prep Kit for Illumina® (New England Biolabs, USA).

5.1.4.2 Sequencing

The constructed amplicon library was sequenced by PE250 using the Illumina Nova 6000 platform. (Guangdong Magigene Biotechnology Co., Ltd. Guangzhou, China)

**5.2. Analyze the process**

**5.2.1 Sequencing data processing**

(1) Paired-end raw reads data filtering

(2) Paired-end clean reads splicing

(3) Raw tags sequence quality filtering

**5.2.2 OTU clustering and species annotation**

(1) Clustering method: OTU, the clustering method was UPARSE

(2) Database：SILVA

(3) Representative sequence species annotation: the representative sequences of each OTU were compared with the SILVA(16S), the default confidence threshold was set to 0.8.

**5.2.3 OTU Statistics**

(1) OTU_Table: based on the above OTU_table after removing singleton OTU, chimera and contaminated OTU, the number of reads and otus contained in samples or groups were counted.

(2) Pan_Core species analysis: R software (V5.1.3) was used to calculate the union number (Pan) and intersection number (Core) of target classification levels in different sample numbers to evaluate the adequacy of sample size.

**5.2.4 Species community analysis**

Species community structure: R software was used to analyze common and endemic species statistics, community composition analysis, and species abundance cluster analysis.

**5.2.5 Alpha Diversity Analysis**

Alpha diversity indices were calculated using the OTU abundance table with default parameters via the usearch - alpha_div tool (V10, http://www.drive5.com/usearch/). The diversity indices (Chao1, Simpson index) were analyzed, and spss software was employed to assess differences in alpha diversity between groups.

**5.2.6 Beta Diversity Analysis**

Principal Coordinate Analysis (PCoA) was conducted using the OTU abundance table with the bray-curtis distance algorithm via the vegan package in R software.

**5.2.7 Species Difference Analysis**

LEfSe analysis was performed using LEfSe software to identify significant species differences between groups based on the OTU abundance table. The non-parametric Kruskal-Wallis (KW) sum-rank test was first employed to detect significant differences in species abundance among groups, followed by the Student's t-test for pairwise comparisons. Linear Discriminant Analysis (LDA) was then used to reduce dimensionality and assess the impact of significant species, with a default LDA Score threshold of 3 to determine biomarkers for each group.

**6. Metabolomics analysis of *D.melanogaster***

**6.1 Sample information**

A total of 17 *Drosophila* intestinal body tissue samples were used for LC-MS metabolomics detection and analysis.

| Name | Number of samples |
| --- | --- |
| Aged | 5 |
| Young | 6 |
| AAL | 6 |

**6.2** **Experimental Information**

**6.2.1. Instruments and reagents**

6.2.1.1. Experimental reagents

| Name | CAS | purity | brand |
| --- | --- | --- | --- |
| Methanol | 67-56-1 | LC-MS | CNW Technologies |
| Acetonitrile | 75-05-8 | LC-MS | CNW Technologies |
| Ammonium acetate | 631-61-8 | LC-MS | SIGMA-ALDRICH |
| Ammonium hydroxide | 1336-21-6 | LC-MS | Fisher Chemical |
| ddH2O |  |  | Watsons |

6.2.1.2. Experimental apparatus

| Instrument | Model number | Brand |
| --- | --- | --- |
| Ultra-high performance liquid phase | Vanquish | Thermo Fisher Scientific |
| High resolution mass spectrometry | Orbitrap Exploris 120 | Thermo Fisher Scientific |
| Centrifuge machine | Heraeus Fresco17 | Thermo Fisher Scientific |
| Electronic balance | BSA124S-CW | Sartorius |
| Ultrasonic ultrasonic apparatus | PS-60AL | Shenzhen REDBANG Electronics Co. LTD |

**6.2.2 Experimental Methods**

6.2.2.1.Metabolites Extraction：

25 mg of sample was weighted to an EP tube, and 500μL extract solution (methanol: acetonitrile: water = 2: 2: 1, with isotopically-labelled internal standard mixture) was added. Then the samples were homogenized at 35 Hz for 4 min and sonicated for 5 min in ice-water bath. The homogenization and sonication cycle were repeated for 3 times. Then the samples were incubated for 1 h at -40 ℃ and centrifuged at 12000 rpm (RCF=13800(×g),R= 8.6cm) for 15 min at 4 ℃. The resulting supernatant was transferred to a fresh glass vial for analysis. The quality control (QC) sample was prepared by mixing an equal aliquot of the supernatants from all of the samples

6.2.2.2 Test on machine

A Vanquish ultra-performance liquid chromatograph (Thermo Fisher Scientific) was used for chromatographic separation of target compounds on a Waters BEH Amide column (2.1 mm × 50 mm, 1.7 μm). The liquid chromatography mobile phase A comprised 25 mmol/L ammonium acetate in water, while phase B consisted of acetonitrile. The sample plate temperature was set to 4 ℃, and the injection volume was 2 µL. The Orbitrap Exploris 120 mass spectrometer was used for primary and secondary mass spectrometry data acquisition, controlled by Xcalibur software (version 4.4, Thermo). Detailed parameters are as follows: sheath gas flow rate, 50 Arb; auxiliary gas flow rate, 15 Arb; capillary temperature, 320 ℃; full MS resolution, 60,000; MS/MS resolution, 30,000; collision energy, 20/30/40 in NCE mode; spray voltage, 3 kV (positive) or -3 kV (negative).

6.2.2.3.*LC-MS/MS* Analysis：

LC-MS/MS analyses were performed using an UHPLC system (Vanquish, Thermo Fisher Scientific) with a Waters BEH Amide column (2.1 mm × 50 mm, 1.7 μm) coupled to Orbitrap Exploris 120 mass spectrometer (Orbitrap MS, Thermo). The mobile phase consisted of 25 mmol/L ammonium acetate and 25 ammonia hydroxide in water（pH = 9.75）(A) and acetonitrile (B). The auto-sampler temperature was 4 ℃, and the injection volume was 2 μL. The Orbitrap Exploris 120 mass spectrometer was used for its ability to acquire MS/MS spectra on information-dependent acquisition (IDA) mode in the control of the acquisition software (Xcalibur, Thermo). In this mode, the acquisition software continuously evaluates the full scan MS spectrum. The ESI source conditions were set as following: sheath gas flow rate as 50 Arb, Aux gas flow rate as 15 Arb, capillary temperature 350 ℃, full MS resolution as 60000, MS/MS resolution as 30000 collision energy as 20/30/40 in NCE mode, spray Voltage as 3 kV (positive) or -3 kV (negative), respectively.

6.2.2.4 Data preprocessing and annotation：

The raw data were converted to the mzXML format using ProteoWizard and processed with an in-house program, which was developed using R and based on XCMS, for peak detection, extraction, alignment, and integration. Then a MS2 database was applied in metabolite annotation. The cutoff for annotation was set at 0.3.

**6.3** **Results of Data analysis**

6.3.1 Pretreatment of raw data

The raw data are shown in the raw data table in the results file. In order to better analyze the data, we prepare and organize the original data in a series of ways. It mainly includes the following steps:

Individual peaks were filtered to remove noise. Based on the relative standard deviation (relative standard deviation, RSD, namely variation coefficient of variation (CV) was used to filter the deviation values.

Individual peaks were filtered. Only peak area data with no more than 50% null values in a single group or no more than 50% null values in all groups were retained.

Missing value recoding is used to simulate missing values in the original data. The numerical simulation method was filled with one-half of the minimum value.

Data were processed by normalization. Internal standard (IS) was used for normalization.

Then, we mapped all the metabolites of this project to authoritative metabolite databases such as KEGG and PubChem.

**6.3.2 Principal Component Analysis (PCA)**

The analysis was carried out by using “fast.prcomp()” function in R software (v.4.2.2) package “gmodels” (v.2.18.1), and the results were then visualized using package “ggpubr” (v0.4.0) through Hiplot Pro (https://hiplot.com.cn/), a comprehensive web service for biomedical data analysis and visualization. The results have been uploaded.

**6.3.3 Differential Metabolite Screening and Volcano Plot**

In this project, the metabolites of the aged group and the young group were compared, and the value standard was *P* < 0.05 of Student's t-test, and FC≥2 or FC≤0.5 to identify meaningful differential metabolites. The plot was generated using R software (v.4.2.2) package “ggpubr” (v0.4.0) and “ggplot2” (v3.4.2) through hiplot pro (https://hiplot.com.cn/), a comprehensive web service for biomedical data analysis and visualization.

**6.3.4 Pathway Analysis**

In MetaboAnalyst 6.0(https://www.metaboanalyst.ca/), select enrichment analysis, input potential compounds screened by AAL, input type: compound names, feature type: metabolites, input type: compound names, feature type: metabolites, pathway based: KEGG. Only use metabolite sets containing at least 2 entries was selected to obtain Results of Enrichment Analysis of potential metabolites. The results have been uploaded.

**6.3.5 Integrated Omics Analysis**

In Magigene platform(http://cloud.magigene.com/), the data of major genera and potential metabolites were input respectively. Correlation analysis method: spearman, clustering method: ward.D, output marker significance, and obtain correlation heat map. Correlation data sheets and *p*-value data sheets have been uploaded.

**Result**

**1.Result for UPLC-MS/MS profile of AAL**


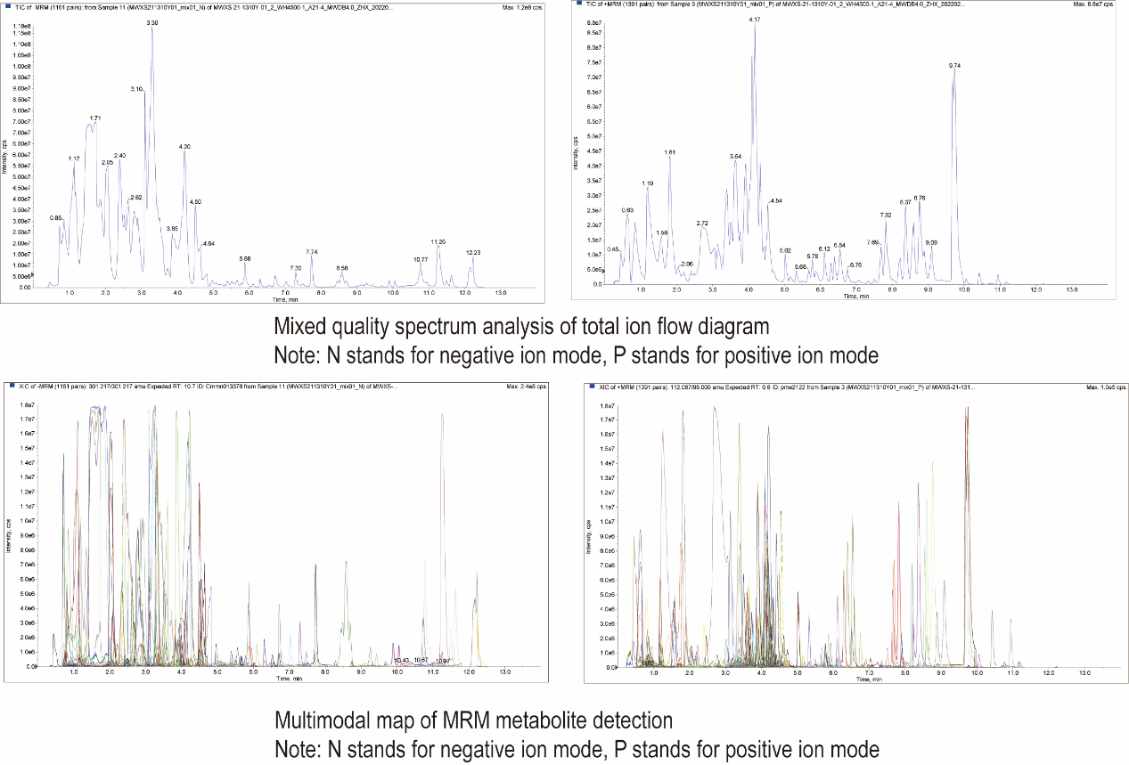


**Figure S2** the UPLC-MS/MS profile of AAL

| **Table S1** the UPLC-MS/MS profile of AAL | | | | | | |
| --- | --- | --- | --- | --- | --- | --- |
| **Formula** | **Ionization model** | **Compounds** | **Class I** | **Class II** | **CAS** | **AAL** |
| **C16H18O9** | **[M-H]-** | **Chlorogenic acid (3-O-Caffeoylquinic acid)*** | **Phenolic acids** | **Phenolic acids** | **327-97-9** | **1.04E+08** |
| **C16H18O8** | **[M-H]-** | **3-O-p-Coumaroylquinic acid*** | **Phenolic acids** | **Phenolic acids** | **87099-71-6** | **1.01E+08** |
| **C21H20O11** | **[M+H]+** | **Quercetin-3-O-rhamnoside(Quercitrin)** | **Flavonoids** | **Flavonols** | **522-12-3** | **93343000** |
| **C16H22O4** | **[M+H]+** | **Diisobutyl phthalate*** | **Phenolic acids** | **Phenolic acids** | **84-69-5** | **81821000** |
| **C16H22O4** | **[M+H]+** | **Dibutyl phthalate*** | **Phenolic acids** | **Phenolic acids** | **84-74-2** | **78609000** |
| **C4H6O4** | **[M-H]-** | **succinic acid** | **Organic acids** | **Organic acids** | **110-15-6** | **50620000** |
| **C18H32O2** | **[M-H]-** | **Linoleic acid** | **Lipids** | **Free fatty acids** | **60-33-3** | **49778000** |
| **C5H5N5** | **[M+H]+** | **Adenine** | **Nucleotides and derivatives** | **Nucleotides and derivatives** | **73-24-5** | **39384000** |
| **C16H18O9** | **[M-H]-** | **Neochlorogenic acid (5-O-Caffeoylquinic acid)*** | **Phenolic acids** | **Phenolic acids** | **906-33-2** | **29582000** |
| **C13H8O8** | **[M-H]-** | **Brevifolin carboxylic acid** | **Phenolic acids** | **Phenolic acids** | **18490-95-4** | **26515000** |
| **C15H16O9** | **[M-H]-** | **Esculetin-7-O-glucoside*** | **Lignans and Coumarins** | **Coumarins** | **-** | **22143000** |
| **C15H16O9** | **[M-H]-** | **Esculin (6,7-DihydroxyCoumarin-6-glucoside)*** | **Lignans and Coumarins** | **Coumarins** | **531-75-9** | **20985000** |
| **C15H16O9** | **[M-H]-** | **Daphnin*** | **Lignans and Coumarins** | **Coumarins** | **486-55-5** | **20571000** |
| **C9H16O4** | **[M-H]-** | **Azelaic acid** | **Organic acids** | **Organic acids** | **123-99-9** | **19637000** |
| **C6H8O7** | **[M-H]-** | **Citric Acid** | **Organic acids** | **Organic acids** | **77-92-9** | **19144000** |
| **C9H11NO2** | **[M+H]+** | **L-Phenylalanine** | **Amino acids and derivatives** | **Amino acids and derivatives** | **63-91-2** | **18394000** |
| **C18H34O5** | **[M-H]-** | **9,10,13-Trihydroxy-11-Octadecenoic Acid*** | **Lipids** | **Free fatty acids** | **29907-57-1** | **18185000** |
| **C27H30O15** | **[M+H]+** | **Quercetin-3,7-Di-O-rhamnoside** | **Flavonoids** | **Flavonols** | **28638-13-3** | **17087000** |
| **C6H8O7** | **[M-H]-** | **Isocitric Acid** | **Organic acids** | **Organic acids** | **320-77-4** | **16585000** |
| **C22H22O11** | **[M+H]+** | **6-C-Methylquercetin-3-O-rhamnoside** | **Flavonoids** | **Flavonols** | **-** | **15776000** |
| **C28H32O16** | **[M+H]+** | **ISOrhamnetin-3-O-glucoside-7-O-rhamnoside*** | **Flavonoids** | **Flavonols** | **-** | **13505000** |
| **C5H9NO2** | **[M+H]+** | **L-Proline** | **Amino acids and derivatives** | **Amino acids and derivatives** | **147-85-3** | **13420000** |
| **C6H13NO2** | **[M+H]+** | **L-Isoleucine*** | **Amino acids and derivatives** | **Amino acids and derivatives** | **73-32-5** | **11087000** |
| **C6H13NO2** | **[M+H]+** | **L-Leucine*** | **Amino acids and derivatives** | **Amino acids and derivatives** | **61-90-5** | **11023000** |
| **C27H30O16** | **[M+H]+** | **Quercetin-7-O-rutinoside*** | **Flavonoids** | **Flavonols** | **147714-62-3** | **10712000** |
| **C6H13NO2** | **[M+H]+** | **L-Norleucine*** | **Amino acids and derivatives** | **Amino acids and derivatives** | **327-57-1** | **10426000** |
| **C9H6O4** | **[M-H]-** | **Esculetin** | **Lignans and Coumarins** | **Coumarins** | **305-01-1** | **9368900** |
| **C16H18O9** | **[M-H]-** | **Cryptochlorogenic acid (4-O-Caffeoylquinic acid)*** | **Phenolic acids** | **Phenolic acids** | **905-99-7** | **9055800** |
| **C4H6O5** | **[M-H]-** | **L-Malic acid** | **Organic acids** | **Organic acids** | **97-67-6** | **9004200** |
| **C22H22O11** | **[M+H]+** | **Diosmetin-7-O-galactoside** | **Flavonoids** | **Flavones** | **-** | **8584100** |
| **C21H20O12** | **[M-H]-** | **Quercetin-7-O-glucoside*** | **Flavonoids** | **Flavonols** | **491-50-9** | **8561100** |
| **C6H12O3** | **[M-H]-** | **2-Hydroxyisocaproic acid** | **Organic acids** | **Organic acids** | **20312-37-2** | **7417800** |
| **C13H16O10** | **[M-H]-** | **5-O-Galloyl-D-hamamelose*** | **Phenolic acids** | **Phenolic acids** | **-** | **7035500** |
| **C18H32O4** | **[M-H]-** | **9-Hydroxy-12-oxo-15(Z)-octadecenoic acid** | **Lipids** | **Free fatty acids** | **-** | **6799500** |
| **C10H8O5** | **[M+H]+** | **Fraxetin (7,8-Dihydroxy-6-methoxycoumarin)*** | **Lignans and Coumarins** | **Coumarins** | **574-84-5** | **6786300** |
| **C11H15N5O4** | **[M+H]+** | **N6-methyladenosine** | **Nucleotides and derivatives** | **Nucleotides and derivatives** | **1867-73-8** | **6668600** |
| **C5H5N5O** | **[M+H]+** | **Guanine** | **Nucleotides and derivatives** | **Nucleotides and derivatives** | **73-40-5** | **6653200** |
| **C9H10O4** | **[M-H]-** | **Methyl 2,4-dihydroxyphenylacetate** | **Phenolic acids** | **Phenolic acids** | **67828-42-6** | **6335100** |
| **C9H10O4** | **[M-H]-** | **Hydroxyphenyllactic acid** | **Phenolic acids** | **Phenolic acids** | **306-23-0** | **6270100** |
| **C21H20O10** | **[M+H]+** | **Genistein-8-C-glucoside** | **Flavonoids** | **Isoflavones** | **66026-80-0** | **5978700** |
| **C14H14O9** | **[M-H]-** | **5-Galloylshikimic acid*** | **Phenolic acids** | **Phenolic acids** | **95719-51-0** | **5903000** |
| **C8H4O3** | **[M+H]+** | **Phthalic anhydride** | **Phenolic acids** | **Phenolic acids** | **85-44-9** | **5799300** |
| **C11H10O5** | **[M+H]+** | **Isofraxidin** | **Lignans and Coumarins** | **Coumarins** | **486-21-5** | **5642800** |
| **C9H10O5** | **[M-H]-** | **syringic acid** | **Phenolic acids** | **Phenolic acids** | **530-57-4** | **5629600** |
| **C8H8O4** | **[M-H]-** | **Vanillic acid** | **Phenolic acids** | **Phenolic acids** | **121-34-6** | **5164600** |
| **C16H18O8** | **[M-H]-** | **1-O-p-Coumaroylquinic acid*** | **Phenolic acids** | **Phenolic acids** | **-** | **5122700** |
| **C20H18O12** | **[M-H]-** | **Myricetin-3-O-arabinoside*** | **Flavonoids** | **Flavonols** | **132679-85-7** | **4738300** |
| **C8H8O5** | **[M-H]-** | **3-O-Methylgallic acid** | **Phenolic acids** | **Phenolic acids** | **3934-84-7** | **4631500** |
| **C16H12O6** | **[M+H]+** | **Diosmetin (5,7,3'-Trihydroxy-4'-methoxyflavone)*** | **Flavonoids** | **Flavones** | **520-34-3** | **4490900** |
| **C20H18O12** | **[M-H]-** | **Myricetin-3-O-xyloside*** | **Flavonoids** | **Flavonols** | **-** | **4307300** |
| **C9H10O3** | **[M-H]-** | **DL-3-Phenyllactic acid** | **Organic acids** | **Organic acids** | **828-01-3** | **4028000** |
| **C6H11NO2** | **[M+H]+** | **Cycloleucine** | **Amino acids and derivatives** | **Amino acids and derivatives** | **52-52-8** | **3994700** |
| **C27H30O15** | **[M+H]+** | **Kaempferol-3-O-rutinoside(Nicotiflorin)*** | **Flavonoids** | **Flavonols** | **17650-84-9** | **3955200** |
| **C27H30O16** | **[M+H]+** | **Quercetin-3-O-neohesperidoside*** | **Flavonoids** | **Flavonols** | **117611-67-3** | **3938600** |
| **C13H16O9** | **[M-H]-** | **Protocatechuic acid-4-O-glucoside*** | **Phenolic acids** | **Phenolic acids** | **-** | **3932600** |
| **C27H30O15** | **[M+H]+** | **Kaempferol-3-O-neohesperidoside*** | **Flavonoids** | **Flavonols** | **32602-81-6** | **3901900** |
| **C15H10O7** | **[M+H]+** | **Quercetin*** | **Flavonoids** | **Flavonols** | **117-39-5** | **3681000** |
| **C8H15NO6** | **[M+H]+** | **N-Acetyl-D-glucosamine*** | **Others** | **Saccharides and Alcohols** | **7512-17-6** | **3567300** |
| **C9H11NO3** | **[M+H]+** | **L-Tyrosine** | **Amino acids and derivatives** | **Amino acids and derivatives** | **60-18-4** | **3554400** |
| **C26H34O11** | **[M-H]-** | **Lariciresinol-4'-O-glucoside*** | **Lignans and Coumarins** | **Lignans** | **143663-00-7** | **3512500** |
| **C27H30O17** | **[M+H]+** | **Myricetin-3-O-galactoside-3'-O-rhamnoside*** | **Flavonoids** | **Flavonols** | **-** | **3461200** |
| **C27H30O16** | **[M-H]-** | **Quercetin-3-O-robinobioside*** | **Flavonoids** | **Flavonols** | **52525-35-6** | **3255000** |
| **C15H14O6** | **[M-H]-** | **5,7,3',4',5'-Pentahydroxyflavan (Tricetiflavan)** | **Flavonoids** | **Flavanols** | **-** | **3110200** |
| **C28H24O16** | **[M+H]+** | **Quercetin-3-O-(6''-O-galloyl)galactoside*** | **Flavonoids** | **Flavonols** | **53171-28-1** | **3063200** |
| **C15H10O5** | **[M+H]+** | **Apigenin; 4',5,7-Trihydroxyflavone** | **Flavonoids** | **Flavones** | **520-36-5** | **3038100** |
| **C5H11NO2** | **[M+H]+** | **L-Valine** | **Amino acids and derivatives** | **Amino acids and derivatives** | **72-18-4** | **3014700** |
| **C6H11NO2** | **[M+H]+** | **DL-Pipecolic Acid** | **Organic acids** | **Organic acids** | **3105-95-1** | **2975300** |
| **C4H6O5** | **[M-H]-** | **3-Dehydro-L-Threonic Acid** | **Others** | **Saccharides and Alcohols** | **-** | **2973800** |
| **C26H28O15** | **[M+H]+** | **Quercetin-3-O-rhamnosyl(1→2)arabinoside** | **Flavonoids** | **Flavonols** | **-** | **2926500** |
| **C5H11N3O** | **[M+H]+** | **4-Guanidinobutanal** | **Others** | **Others** | **-** | **2921900** |
| **C11H15N5O4** | **[M+H]+** | **2'-O-Methyladenosine** | **Nucleotides and derivatives** | **Nucleotides and derivatives** | **2140-79-6** | **2780000** |
| **C14H17N5O8** | **[M+H]+** | **Succinyladenosine** | **Nucleotides and derivatives** | **Nucleotides and derivatives** | **4542-23-8** | **2497200** |
| **C15H10O7** | **[M+H]+** | **Robinetin** | **Flavonoids** | **Flavonols** | **490-31-3** | **2489000** |
| **C16H35NO2** | **[M+H]+** | **Hexadecylsphingosine** | **Lipids** | **Sphingolipids** | **-** | **2468400** |
| **C11H20O4** | **[M-H]-** | **Undecanedioic acid** | **Lipids** | **Free fatty acids** | **1852-04-6** | **2369600** |
| **C15H12O5** | **[M-H]-** | **Naringenin (5,7,4'-Trihydroxyflavanone)** | **Flavonoids** | **Flavanones** | **480-41-1** | **2360400** |
| **C9H11NO3** | **[M+H]+** | **N-(2-Hydroxy-4-methoxyphenyl)acetamide** | **Alkaloids** | **Phenolamine** | **58469-06-0** | **2281800** |
| **C9H8O3** | **[M-H]-** | **α-Hydroxycinnamic acid*** | **Phenolic acids** | **Phenolic acids** | **5801-57-0** | **2061500** |
| **C27H30O16** | **[M-H]-** | **Quercetin-3-O-rutinoside (Rutin)*** | **Flavonoids** | **Flavonols** | **153-18-4** | **2058600** |
| **C9H8O3** | **[M-H]-** | **2-Hydroxycinnamic acid*** | **Phenolic acids** | **Phenolic acids** | **583-17-5** | **1967200** |
| **C24H20O9** | **[M-H]-** | **Catechin-(7,8-bc)-4β-(3,4-dihydroxyphenyl)-dihydro-2-(3H)-one** | **Flavonoids** | **Flavanols** | **-** | **1899300** |
| **C28H24O16** | **[M+H]+** | **Quercetin-3-O-(2''-O-galloyl)glucoside*** | **Flavonoids** | **Flavonols** | **69624-79-9** | **1886000** |
| **C20H20O14** | **[M+H]+** | **2,3-Di-O-Galloyl-β-D-glucose*** | **Phenolic acids** | **Phenolic acids** | **-** | **1872200** |
| **C16H32O3** | **[M-H]-** | **2-Hydroxyhexadecanoic acid** | **Organic acids** | **Organic acids** | **764-67-0** | **1829100** |
| **C9H11NO3** | **[M+H]+** | **4-Hydroxy-5-(2-oxo-1-pyrrolidinyl)benzoic acid** | **Alkaloids** | **Pyrrole alkaloids** | **-** | **1814100** |
| **C5H8O5** | **[M-H]-** | **L-Citramalic acid*** | **Organic acids** | **Organic acids** | **-** | **1684100** |
| **C8H8O4** | **[M-H]-** | **Protocatechuic Acid Methyl Ester** | **Phenolic acids** | **Phenolic acids** | **2150-43-8** | **1672000** |
| **C11H10O5** | **[M+H]+** | **Fraxidin (8-Hydroxy-6,7-dimethoxycoumarin)** | **Lignans and Coumarins** | **Coumarins** | **525-21-3** | **1619800** |
| **C5H8O5** | **[M-H]-** | **2-Hydroxyglutaric Acid** | **Organic acids** | **Organic acids** | **13095-48-2** | **1510100** |
| **C15H12O5** | **[M+H]+** | **Naringenin chalcone** | **Flavonoids** | **Chalcones** | **73692-50-9** | **1477500** |
| **C18H35NO** | **[M+H]+** | **Oleamide (9-Octadecenamide)** | **Lipids** | **Free fatty acids** | **301-02-0** | **1457300** |
| **C5H8O5** | **[M-H]-** | **2-Dehydro-3-deoxy-L-arabinonate** | **Others** | **Saccharides and Alcohols** | **-** | **1436500** |
| **C16H12O7** | **[M+H]+** | **ISOrhamnetin** | **Flavonoids** | **Flavonols** | **480-19-3** | **1413300** |
| **C5H8O5** | **[M-H]-** | **3-Methylmalic acid*** | **Organic acids** | **Organic acids** | **152204-30-3** | **1397700** |
| **C18H30O4** | **[M-H]-** | **13(s)-hydroperoxy-(9z,11e,15z)-octadecatrienoic acid** | **Lipids** | **Free fatty acids** | **67597-26-6** | **1385400** |
| **C21H20O10** | **[M+H]+** | **Apigenin-7-O-glucoside(Cosmosiin)** | **Flavonoids** | **Flavones** | **578-74-5** | **1384400** |
| **C4H8O5** | **[M-H]-** | **D-Threonic Acid** | **Others** | **Saccharides and Alcohols** | **-** | **1279900** |
| **C15H10O8** | **[M+H]+** | **Myricetin** | **Flavonoids** | **Flavonols** | **529-44-2** | **1267600** |
| **C15H10O6** | **[M+H]+** | **Kaempferol (3,5,7,4'-Tetrahydroxyflavone)** | **Flavonoids** | **Flavonols** | **520-18-3** | **1190200** |
| **C9H10O4** | **[M-H]-** | **Vanillic acid methyl ester** | **Phenolic acids** | **Phenolic acids** | **3943-74-6** | **1155000** |
| **C10H12O4** | **[M-H]-** | **Dihydroferulic Acid** | **Phenolic acids** | **Phenolic acids** | **1135-23-5** | **1075600** |
| **C21H20O10** | **[M+H]+** | **Genistein-7-O-galactoside** | **Flavonoids** | **Isoflavones** | **-** | **1055200** |
| **C5H5N5** | **[M+H]+** | **2-Aminopurine** | **Nucleotides and derivatives** | **Nucleotides and derivatives** | **452-06-2** | **1028300** |
| **C14H14O9** | **[M-H]-** | **3-Galloylshikimic acid*** | **Phenolic acids** | **Phenolic acids** | **110082-91-2** | **985530** |
| **C21H24O11** | **[M-H]-** | **Epicatechin-3'-O-β-D-glucopyranoside*** | **Flavonoids** | **Flavanols** | **-** | **950080** |
| **C9H10O5** | **[M-H]-** | **Gallic Acid Ethyl Ester; Ethyl gallate** | **Phenolic acids** | **Phenolic acids** | **831-61-8** | **948880** |
| **C21H22O10** | **[M-H]-** | **Naringenin-7-O-glucoside (Prunin)*** | **Flavonoids** | **Flavanones** | **529-55-5** | **938030** |
| **C13H16O10** | **[M-H]-** | **2-O-Galloyl-D-glucose*** | **Phenolic acids** | **Phenolic acids** | **-** | **910840** |
| **C16H30O4** | **[M-H]-** | **Hexadecanedioic acid** | **Lipids** | **Free fatty acids** | **505-54-4** | **854710** |
| **C6H12O6** | **[M-H]-** | **D-Glucose*** | **Others** | **Saccharides and Alcohols** | **14431-43-7** | **838390** |
| **C23H24O13** | **[M+H]+** | **Syringetin-7-O-glucoside** | **Flavonoids** | **Flavonols** | **-** | **835840** |
| **C8H6O4** | **[M-H]-** | **Phthalic acid*** | **Phenolic acids** | **Phenolic acids** | **88-99-3** | **805690** |
| **C6H12O6** | **[M-H]-** | **D-Galactose*** | **Others** | **Saccharides and Alcohols** | **59-23-4** | **799250** |
| **C6H12O6** | **[M-H]-** | **D-Fructose*** | **Others** | **Saccharides and Alcohols** | **7660-25-5** | **796250** |
| **C15H14O7** | **[M-H]-** | **Epigallocatechin** | **Flavonoids** | **Flavanols** | **970-74-1** | **770130** |
| **C16H18O8** | **[M-H]-** | **4-O-p-Coumaroylquinic acid*** | **Phenolic acids** | **Phenolic acids** | **32451-86-8** | **712420** |
| **C10H14N5O8P** | **[M+H]+** | **Guanosine 5'-monophosphate** | **Nucleotides and derivatives** | **Nucleotides and derivatives** | **85-32-5** | **695080** |
| **C10H12O4** | **[M-H]-** | **3,4-Dimethoxyphenyl acetic acid** | **Phenolic acids** | **Phenolic acids** | **93-40-3** | **690630** |
| **C6H8O6** | **[M-H]-** | **D-Glucurono-6,3-lactone** | **Others** | **Saccharides and Alcohols** | **32449-92-6** | **684300** |
| **C5H6O4** | **[M-H]-** | **Methylenesuccinic acid** | **Organic acids** | **Organic acids** | **97-65-4** | **673970** |
| **C12H24O2** | **[M-H]-** | **Dodecanoic acid (Lauric acid)** | **Lipids** | **Free fatty acids** | **143-07-7** | **647950** |
| **C9H12O4** | **[M+H]+** | **Antiarol; 3,4,5-Trimethoxyphenol** | **Phenolic acids** | **Phenolic acids** | **642-71-7** | **585350** |
| **C15H18O9** | **[M-H]-** | **6-O-Caffeoyl-D-glucose*** | **Phenolic acids** | **Phenolic acids** | **-** | **582200** |
| **C6H12O6** | **[M-H]-** | **D-Mannose*** | **Others** | **Saccharides and Alcohols** | **3458-28-4** | **556870** |
| **C12H24O3** | **[M-H]-** | **12-Hydroxydodecanoic acid** | **Lipids** | **Free fatty acids** | **505-95-3** | **541410** |
| **C4H6O6** | **[M-H]-** | **L-Tartaric acid** | **Organic acids** | **Organic acids** | **87-69-4** | **527570** |
| **C6H14O6** | **[M-H]-** | **D-Sorbitol*** | **Others** | **Saccharides and Alcohols** | **50-70-4** | **524150** |
| **C14H22O** | **[M-H]-** | **2,4-Di-Tert-Butylphenol*** | **Phenolic acids** | **Phenolic acids** | **96-76-4** | **522590** |
| **C14H22O** | **[M-H]-** | **2,6-Di-tert-butylphenol*** | **Phenolic acids** | **Phenolic acids** | **128-39-2** | **520370** |
| **C28H24O15** | **[M+H]+** | **Kaempferol-3-O-(6''-galloyl)galactoside*** | **Flavonoids** | **Flavonols** | **-** | **512450** |
| **C10H12N5O6P** | **[M-H]-** | **Cyclic 3',5'-Adenylic acid** | **Nucleotides and derivatives** | **Nucleotides and derivatives** | **60-92-4** | **473000** |
| **C20H18O6** | **[M+H]+** | **8-Prenylkaempferol** | **Flavonoids** | **Flavones** | **28610-31-3** | **472260** |
| **C12H16O3** | **[M+H]+** | **alpha-Asarone*** | **Others** | **Others** | **2883-98-9** | **466780** |
| **C10H10O4** | **[M-H]-** | **Isoferulic Acid*** | **Phenolic acids** | **Phenolic acids** | **537-73-5** | **452490** |
| **C10H10O4** | **[M-H]-** | **Ferulic acid*** | **Phenolic acids** | **Phenolic acids** | **537-98-4** | **434440** |
| **C10H12N5O7P** | **[M-H]-** | **Guanosine 3',5'-cyclic monophosphate** | **Nucleotides and derivatives** | **Nucleotides and derivatives** | **7665-99-8** | **431360** |
| **C28H32O16** | **[M+H]+** | **ISOrhamnetin-3-O-galactoide-7-O-rhamnoside*** | **Flavonoids** | **Flavonols** | **-** | **430820** |
| **C9H6O4** | **[M+H]+** | **3,7-Dihydroxychromen-4-one** | **Others** | **Others** | **-** | **427740** |
| **C11H15N5O3S** | **[M+H]+** | **5'-Deoxy-5'-(methylthio)adenosine** | **Nucleotides and derivatives** | **Nucleotides and derivatives** | **2457-80-9** | **402300** |
| **C10H10O3** | **[M-H]-** | **3,4-Methylenedioxy cinnamyl alcohol** | **Lignans and Coumarins** | **Lignans** | **58095-76-4** | **402040** |
| **C6H10O7** | **[M-H]-** | **D-Glucoronic acid*** | **Others** | **Saccharides and Alcohols** | **-** | **400580** |
| **C27H24O18** | **[M-H]-** | **1,2,3-Tri-O-galloyl-D-glucose*** | **Phenolic acids** | **Phenolic acids** | **-** | **398240** |
| **C6H10O8** | **[M-H]-** | **D-Saccharic acid*** | **Others** | **Saccharides and Alcohols** | **87-73-0** | **371760** |
| **C11H18N2O2** | **[M+H]+** | **Cyclo(D-Leu-L-Pro)** | **Amino acids and derivatives** | **Amino acids and derivatives** | **36238-67-2** | **367550** |
| **C20H20O14** | **[M+H]+** | **1,6-di-O-galloyl-β-D-glucose*** | **Phenolic acids** | **Phenolic acids** | **-** | **362690** |
| **C8H9N** | **[M+H]+** | **N-Benzylmethylene isomethylamine** | **Alkaloids** | **Alkaloids** | **-** | **334350** |
| **C21H42NO7P** | **[M+H]+** | **LysoPE 16:1(2n isomer)** | **Lipids** | **LPE** | **-** | **331610** |
| **C11H18N2O2** | **[M+H]+** | **Cyclo(Pro-Leu)** | **Amino acids and derivatives** | **Amino acids and derivatives** | **5654-86-4** | **314380** |
| **C6H12O7** | **[M-H]-** | **Gluconic acid** | **Others** | **Saccharides and Alcohols** | **526-95-4** | **312980** |
| **C6H14O6** | **[M-H]-** | **D-Mannitol*** | **Others** | **Saccharides and Alcohols** | **69-65-8** | **310160** |
| **C12H22O11** | **[M-H]-** | **Galactinol*** | **Others** | **Saccharides and Alcohols** | **3687-64-7** | **296900** |
| **C11H13NO3** | **[M-H]-** | **N-Acetyl-L-phenylalanine** | **Amino acids and derivatives** | **Amino acids and derivatives** | **2018-61-3** | **281740** |
| **C15H10O6** | **[M+H]+** | **2'-Hydroxygenistein*** | **Flavonoids** | **Isoflavones** | **1156-78-1** | **267940** |
| **C6H10O7** | **[M-H]-** | **D-Galacturonic acid*** | **Others** | **Saccharides and Alcohols** | **685-73-4** | **262290** |
| **C21H20O10** | **[M+H]+** | **4',5-DihydroxyISOflavone-7-O-galactoside** | **Flavonoids** | **Isoflavones** | **-** | **256360** |
| **C6H9N3O2** | **[M+H]+** | **L-Histidine** | **Amino acids and derivatives** | **Amino acids and derivatives** | **71-00-1** | **247770** |
| **C28H24O15** | **[M+H]+** | **Kaempferol-3-O-(6''-galloyl)glucoside*** | **Flavonoids** | **Flavonols** | **56317-05-6** | **241970** |
| **C6H10O8** | **[M-H]-** | **D-Galactaric acid*** | **Others** | **Saccharides and Alcohols** | **526-99-8** | **240790** |
| **C15H10O6** | **[M+H]+** | **Luteolin (5,7,3',4'-Tetrahydroxyflavone)*** | **Flavonoids** | **Flavones** | **491-70-3** | **236290** |
| **C28H24O15** | **[M+H]+** | **Kaempferol-3-O-(2''-galloyl)galactoside*** | **Flavonoids** | **Flavonols** | **-** | **229980** |
| **C28H24O16** | **[M+H]+** | **Quercetin-3-O-(6''-O-galloyl)glucoside*** | **Flavonoids** | **Flavonols** | **56316-75-7** | **229970** |
| **C17H14O8** | **[M-H]-** | **Syringetin** | **Flavonoids** | **Flavonols** | **4423-37-4** | **229770** |
| **C6H13NO2** | **[M+H]+** | **6-Deoxyfagomine** | **Alkaloids** | **Piperidine alkaloids** | **197449-09-5** | **222960** |
| **C7H13NO3** | **[M-H]-** | **5-Acetamidopentanoic Acid** | **Organic acids** | **Organic acids** | **1072-10-2** | **216020** |
| **C12H22O11** | **[M-H]-** | **D-Maltose*** | **Others** | **Saccharides and Alcohols** | **133-99-3** | **208440** |
| **C23H24O12** | **[M-H]-** | **Tricin-5-O-Glucoside*** | **Flavonoids** | **Flavones** | **32769-00-9** | **196260** |
| **C7H6O2** | **[M-H]-** | **4-Hydroxybenzaldehyde** | **Phenolic acids** | **Phenolic acids** | **123-08-0** | **194550** |
| **C9H7NO** | **[M+H]+** | **Indole-3-carboxaldehyde** | **Alkaloids** | **Plumerane** | **487-89-8** | **188040** |
| **C8H8O** | **[M+H]+** | **(S)-2-Phenyloxirane** | **Others** | **Others** | **20780-54-5** | **181170** |
| **C18H24O** | **[M-H]-** | **Bakuchiol** | **Terpenoids** | **Monoterpenoids** | **10309-37-2** | **178090** |
| **C18H34O4** | **[M-H]-** | **Hydroxy ricinoleic acid** | **Lipids** | **Free fatty acids** | **-** | **169090** |
| **C18H30O2** | **[M-H]-** | **Crepenynic acid** | **Lipids** | **Free fatty acids** | **2277-31-8** | **165790** |
| **C18H30O2** | **[M-H]-** | **γ-Linolenic Acid** | **Lipids** | **Free fatty acids** | **506-26-3** | **159900** |
| **C12H22O11** | **[M-H]-** | **Isomaltulose*** | **Others** | **Saccharides and Alcohols** | **13718-94-0** | **156280** |
| **C28H24O15** | **[M+H]+** | **Kaempferol-3-O-(2''-galloyl)glucoside*** | **Flavonoids** | **Flavonols** | **76343-90-3** | **152980** |
| **C29H26O15** | **[M-H]-** | **1,6-Di-O-galloyl-2-O-Cinnamoyl-β-D-glucose*** | **Phenolic acids** | **Phenolic acids** | **94356-17-9** | **150260** |
| **C29H26O15** | **[M-H]-** | **1,6-Di-O-galloyl-3-O-Cinnamoyl-β-D-glucose*** | **Phenolic acids** | **Phenolic acids** | **-** | **142880** |
| **C13H24O4** | **[M-H]-** | **Tridecanedioic acid** | **Lipids** | **Free fatty acids** | **505-52-2** | **142700** |
| **C5H10O5** | **[M-H]-** | **D-Arabinose** | **Others** | **Saccharides and Alcohols** | **10323-20-3** | **141680** |
| **C12H24N2O3** | **[M+H]+** | **L-Leucyl-L-Leucine** | **Amino acids and derivatives** | **Amino acids and derivatives** | **3303-31-9** | **133620** |
| **C11H12N2O2** | **[M+H]+** | **1-Methoxy-indole-3-acetamide** | **Alkaloids** | **Plumerane** | **-** | **132320** |
| **C12H22O11** | **[M-H]-** | **D-Sucrose*** | **Others** | **Saccharides and Alcohols** | **57-50-1** | **132110** |
| **C8H10O2** | **[M-H]-** | **Tyrosol** | **Phenolic acids** | **Phenolic acids** | **501-94-0** | **120370** |
| **C28H24O16** | **[M+H]+** | **Quercetin-3-O-(2''-O-galloyl)galactoside*** | **Flavonoids** | **Flavonols** | **53209-27-1** | **118580** |
| **C6H12O3** | **[M-H]-** | **6-Hydroxyhexanoic acid** | **Organic acids** | **Organic acids** | **1191-25-9** | **116490** |
| **C8H15NO6** | **[M+H]+** | **N-Acetyl-D-galactosamine*** | **Others** | **Saccharides and Alcohols** | **1811-31-0** | **106190** |
| **C21H40O4** | **[M+H]+** | **1-Oleoyl-Sn-Glycerol** | **Lipids** | **Glycerol ester** | **129784-87-8** | **100520** |
| **C41H32O26** | **[M-H]-** | **1,2,3,4,6-pen-O-galloyl-β-D-glucose*** | **Phenolic acids** | **Phenolic acids** | **-** | **99662** |
| **C12H22O11** | **[M-H]-** | **Melibiose*** | **Others** | **Saccharides and Alcohols** | **585-99-9** | **96588** |
| **C15H10O4** | **[M+H]+** | **Chrysin** | **Flavonoids** | **Flavones** | **480-40-0** | **95154** |
| **C20H24O6** | **[M+H]+** | **(7R,8S)-Dihydrodehydrodiconiferyl alcohol** | **Lignans and Coumarins** | **Lignans** | **126253-41-6** | **93220** |
| **C22H26O8** | **[M-H]-** | **syringaresinol** | **Lignans and Coumarins** | **Lignans** | **21453-71-4** | **91567** |
| **C8H11NO** | **[M+H]+** | **L-Tyramine** | **Amino acids and derivatives** | **Amino acids and derivatives** | **51-67-2** | **89771** |
| **C15H18O9** | **[M-H]-** | **1-O-Caffeoyl-β-D-glucose*** | **Phenolic acids** | **Phenolic acids** | **14364-08-0** | **86778** |
| **C12H22O11** | **[M-H]-** | **D-(+)-Cellobiose*** | **Others** | **Saccharides and Alcohols** | **528-50-7** | **78697** |
| **C12H22O11** | **[M-H]-** | **D-Trehalose*** | **Others** | **Saccharides and Alcohols** | **99-20-7** | **77859** |
| **C6H13O9P** | **[M-H]-** | **D-Glucose 6-phosphate*** | **Others** | **Saccharides and Alcohols** | **56-73-5** | **73573** |
| **C15H20O9** | **[M-H]-** | **Syringic acid 4-O-rhamnoside** | **Lignans and Coumarins** | **Lignans** | **-** | **70784** |
| **C14H20N2O3** | **[M+H]+** | **L-Valyl-L-Phenylalanine** | **Amino acids and derivatives** | **Amino acids and derivatives** | **3918-92-1** | **67801** |
| **C5H10O3** | **[M-H]-** | **2-Hydroxy-2-methylbutyric acid** | **Organic acids** | **Organic acids** | **3739-30-8** | **67744** |
| **C13H18O6** | **[M+H]+** | **Phenyl-β-D-glucopyranoside** | **Others** | **Others** | **-** | **63917** |
| **C18H36O5** | **[M-H]-** | **9,10,18-Trihydroxystearic acid** | **Lipids** | **Free fatty acids** | **496-86-6** | **61718** |
| **C12H22O4** | **[M-H]-** | **Dodecanedioic aicd** | **Lipids** | **Free fatty acids** | **693-23-2** | **56762** |
| **C6H13O9P** | **[M-H]-** | **Glucose-1-phosphate*** | **Others** | **Saccharides and Alcohols** | **59-56-3** | **54470** |
| **C10H14O2** | **[M-H]-** | **9-hydroxythymol** | **Phenolic acids** | **Phenolic acids** | **-** | **48696** |
| **C11H9O2N** | **[M+H]+** | **3-amino-2-naphthoic acid** | **Alkaloids** | **Alkaloids** | **-** | **48029** |
| **C7H7NO2** | **[M+H]+** | **Nicotinic Acid Methyl Ester(Methyl Nicotinate)** | **Alkaloids** | **Pyridine alkaloids** | **93-60-7** | **47745** |
| **C21H38O4** | **[M+H]+** | **1-Linoleoylglycerol*** | **Lipids** | **Glycerol ester** | **2277-28-3** | **46915** |
| **C5H7NO3** | **[M-H]-** | **5-Oxo-L-Proline** | **Amino acids and derivatives** | **Amino acids and derivatives** | **98-79-3** | **44936** |
| **C5H6O5** | **[M-H]-** | **2-Methyl-3-oxosuccinic acid** | **Organic acids** | **Organic acids** | **-** | **44636** |
| **C18H32O2** | **[M-H]-** | **(9Z,11E)-Octadecadienoic acid** | **Lipids** | **Free fatty acids** | **2540-56-9** | **31315** |
| **C15H22O5** | **[M-H]-** | **Octyl gallate** | **Phenolic acids** | **Phenolic acids** | **1034-01-1** | **28555** |
| **C6H14N2O2** | **[M+H]+** | **L-Lysine** | **Amino acids and derivatives** | **Amino acids and derivatives** | **56-87-1** | **26338** |
| **C33H56O14** | **[M+H]+** | **2-α-Linolenoyl-glycerol-1,3-di-O-glucoside*** | **Lipids** | **Glycerol ester** | **-** | **25257** |
| **C8H15NO6** | **[M+H]+** | **N-Acetyl-D-mannosamine*** | **Others** | **Saccharides and Alcohols** | **7772-94-3** | **24311** |
| **C33H56O14** | **[M+H]+** | **1-α-Linolenoyl-glycerol-2,3-di-O-glucoside*** | **Lipids** | **Glycerol ester** | **-** | **23920** |
| **C12H16O3** | **[M+H]+** | **Beta-asarone*** | **Others** | **Others** | **5273-86-9** | **22319** |
| **C27H46O9** | **[M+H]+** | **1-α-Linolenoyl-glycerol-3-O-glucoside*** | **Lipids** | **Glycerol ester** | **-** | **18517** |
| **C27H30O15** | **[M+H]+** | **ISOrhamnetin-3-O-arabinoside-7-O-rhamnoside** | **Flavonoids** | **Flavonols** | **-** | **18313** |
| **C18H37NO** | **[M+H]+** | **Stearamide** | **Alkaloids** | **Alkaloids** | **124-26-5** | **17973** |
| **C19H38O4** | **[M+H]+** | **Monopalmitin** | **Lipids** | **Glycerol ester** | **542-44-9** | **9431.7** |
| **C33H58O14** | **[M+H]+** | **1-Linoleoylglycerol-2,3-di-O-glucoside*** | **Lipids** | **Glycerol ester** | **-** | **8433.1** |
| **C33H58O14** | **[M+H]+** | **2-Linoleoylglycerol-1,3-di-O-glucoside*** | **Lipids** | **Glycerol ester** | **-** | **7613.3** |
| **C33H58O14** | **[M+H]+** | **1-Linoleoyl-sn-glycerol-diglucoside*** | **Lipids** | **Free fatty acids** | **-** | **6716.1** |
| **C21H38O4** | **[M+H]+** | **2-Linoleoylglycerol*** | **Lipids** | **Glycerol ester** | **3443-82-1** | **6649.8** |
| **C10H18O4** | **[M-H]-** | **Sebacate** | **Organic acids** | **Organic acids** | **111-20-6** | **6209.8** |

**2.Result for standard curve diagram**

**
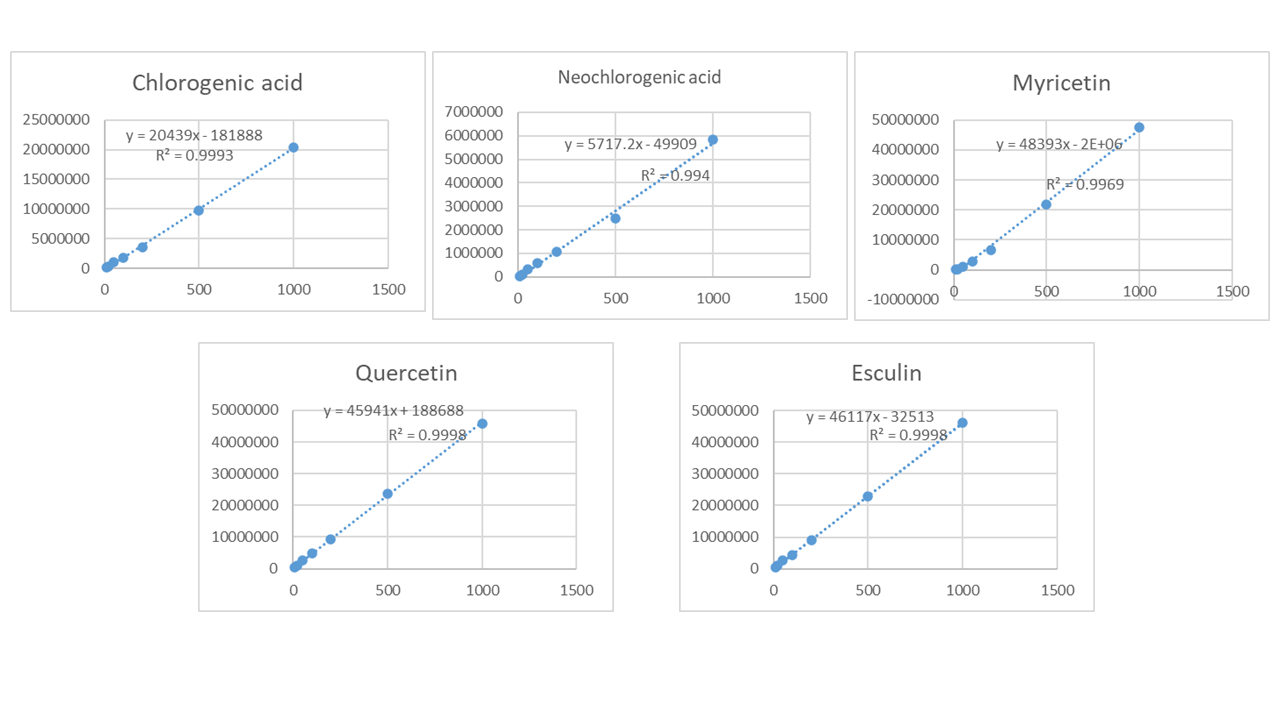
****Figure S3** Standard curve of standard material

**3. Survival analysis of *Drosophila* at different conditions**

| **Table S2** *Drosophila* survival curves | | | | |
| --- | --- | --- | --- | --- |
| Name | Amount | Mean life span(days) | Median life span(days) | Maximum life span(days) |
| Control♀ | 168 | 26.36 | 25 | 53 |
| 0.5%AAL♀ | 182 | 28.86 | 27 | 53 |
| 1%AAL♀ | 179 | 33.77 | 34 | 62 |
| 2%AAL♀ | 172 | 26.23 | 23 | 58 |
| 4%AAL♀ | 160 | 25.57 | 22 | 55 |
| MS♀ | 176 | 28.4 | 26 | 51 |
| Control♂ | 163 | 23.61 | 18 | 44 |
| 0.5%AAL♂ | 173 | 27.2 | 20 | 50 |
| 1%AAL♂ | 172 | 29.06 | 22 | 51 |
| 2%AAL♂ | 178 | 22.38 | 16 | 43 |
| 4%AAL♂ | 160 | 21.8 | 15 | 38 |
| MS♂ | 176 | 26.83 | 20 | 49 |

| **Table S3** Comparison of *Drosophila* survival rates; | | |
| --- | --- | --- |
| Condition | *p*-value | corrected *p*-value |
| C♀ vs AAL0.5%♀ | 0.0171 | 0.0853 |
| C♀ vs AAL1%♀ | 0 | 0 |
| C♀ vs AAL2%♀ | 0.5394 | 1 |
| C♀ vs AAL4%♀ | 0.685 | 1 |
| C♀ vs MS♀ | 0.0801 | 0.4006 |
| C♂ vs AAL0.5%♂ | 0.000038 | 0.0002 |
| C♂ vs AAL1%♂ | 2.00E-08 | 9.90E-08 |
| C♂ vs AAL2%♂ | 0.1489 | 0.7443 |
| C♂ vs AAL4%♂ | 0.0416 | 0.2078 |
| C♂ vs MS♂ | 0.0003 | 0.0016 |

**4. α diversity analysis
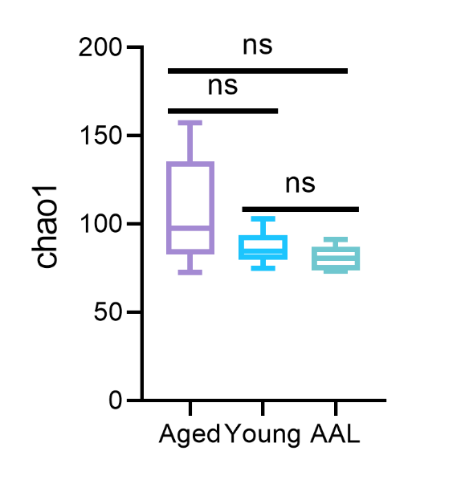
**

**FigureS4**. Chao1 index

**5. Lefse analysis**


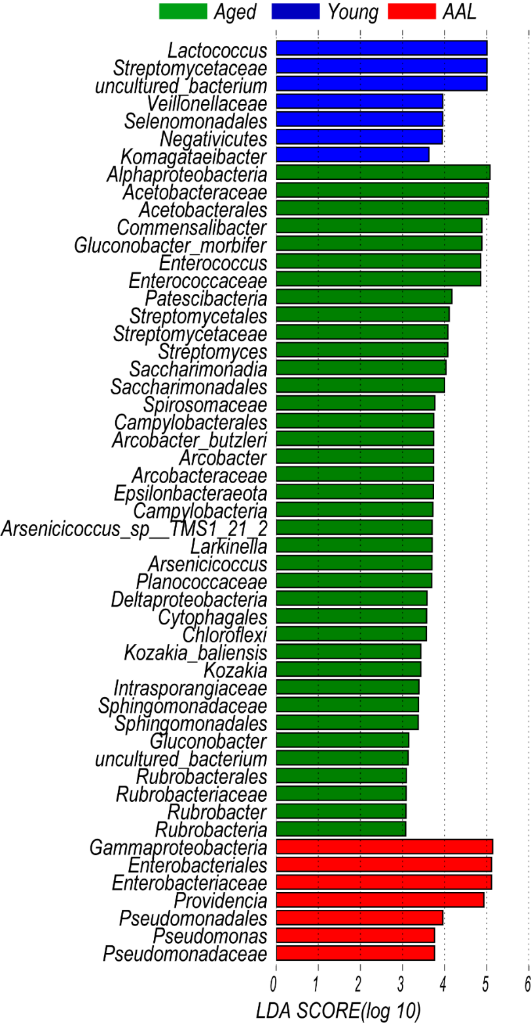


**Figure S5**. Lefse analysis

The figure represents species with significantly different abundances in different groups under conditions greater than the set LDA value, which is set to 2 by default, columns

The length of the plot represents the impact size of the different species

**6. QC sample quality control chart**

**Figure
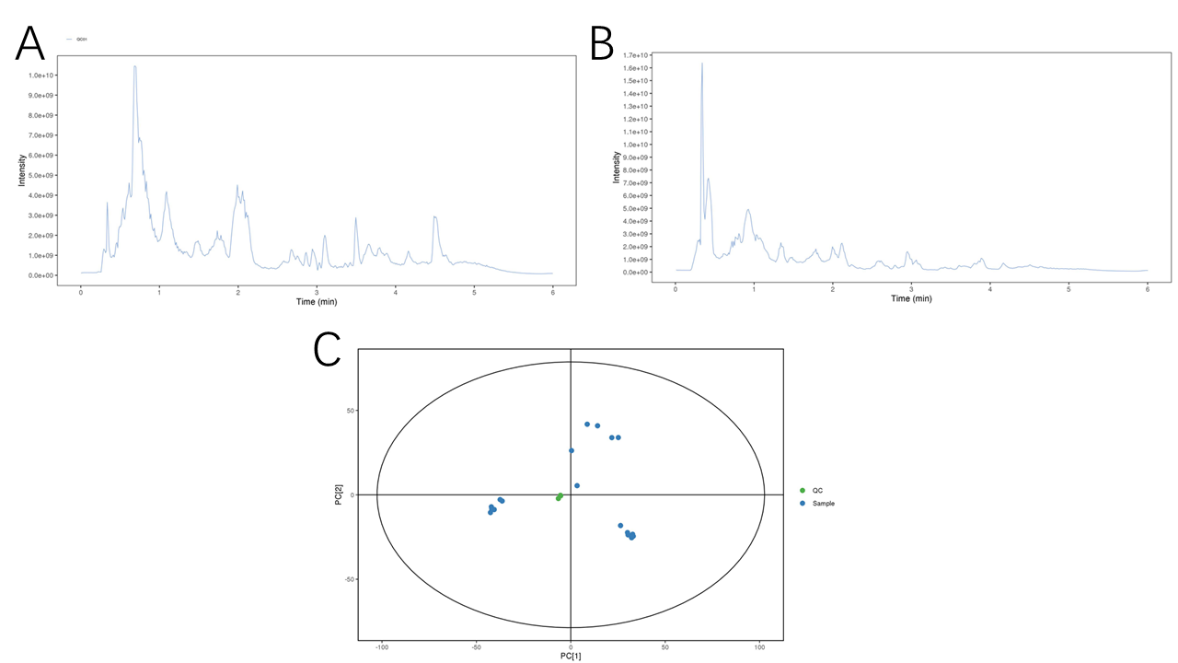
 S6** QC sample quality control chart

A, the positive ion mode TIC of QC samples was detected by UHPLC-OE-MS;B, the negative ion mode TIC of QC samples was detected by UHPLC-OE-MS;C, PCA score plot. Green points are QC samples, and blue points are formal experimental samples.
